# Pseudoscorpion mitochondria show rearranged genes and genome-wide reductions of RNA gene sizes and inferred structures, yet typical nucleotide composition bias

**DOI:** 10.1186/1471-2148-12-31

**Published:** 2012-03-12

**Authors:** Sergey Ovchinnikov, Susan E Masta

**Affiliations:** 1 Department of Biology, Portland State University, P.O. Box 751, Portland, OR 97207, USA

**Keywords:** Chelicerata, Arachnida, Nucleotide skew, tRNA secondary structure, LSU rRNA secondary structure, Genome rearrangements

## Abstract

**Background:**

Pseudoscorpions are chelicerates and have historically been viewed as being most closely related to solifuges, harvestmen, and scorpions. No mitochondrial genomes of pseudoscorpions have been published, but the mitochondrial genomes of some lineages of Chelicerata possess unusual features, including short rRNA genes and tRNA genes that lack sequence to encode arms of the canonical cloverleaf-shaped tRNA. Additionally, some chelicerates possess an atypical guanine-thymine nucleotide bias on the major coding strand of their mitochondrial genomes.

**Results:**

We sequenced the mitochondrial genomes of two divergent taxa from the chelicerate order Pseudoscorpiones. We find that these genomes possess unusually short tRNA genes that do not encode cloverleaf-shaped tRNA structures. Indeed, in one genome, all 22 tRNA genes lack sequence to encode canonical cloverleaf structures. We also find that the large ribosomal RNA genes are substantially shorter than those of most arthropods. We inferred secondary structures of the LSU rRNAs from both pseudoscorpions, and find that they have lost multiple helices. Based on comparisons with the crystal structure of the bacterial ribosome, two of these helices were likely contact points with tRNA T-arms or D-arms as they pass through the ribosome during protein synthesis.

The mitochondrial gene arrangements of both pseudoscorpions differ from the ancestral chelicerate gene arrangement. One genome is rearranged with respect to the location of protein-coding genes, the small rRNA gene, and at least 8 tRNA genes. The other genome contains 6 tRNA genes in novel locations. Most chelicerates with rearranged mitochondrial genes show a genome-wide reversal of the CA nucleotide bias typical for arthropods on their major coding strand, and instead possess a GT bias. Yet despite their extensive rearrangement, these pseudoscorpion mitochondrial genomes possess a CA bias on the major coding strand. Phylogenetic analyses of all 13 mitochondrial protein-coding gene sequences consistently yield trees that place pseudoscorpions as sister to acariform mites.

**Conclusion:**

The well-supported phylogenetic placement of pseudoscorpions as sister to Acariformes differs from some previous analyses based on morphology. However, these two lineages share multiple molecular evolutionary traits, including substantial mitochondrial genome rearrangements, extensive nucleotide substitution, and loss of helices in their inferred tRNA and rRNA structures.

## Background

Pseudoscorpions comprise an ancient order of arachnids, with fossils dating to the Devonian [1]. Other orders of arachnids, such as spiders and scorpions, have unusual mitochondrial genomic features, including reduced tRNA genes and reversed patterns of nucleotide composition, when compared both with other chelicerates and with arthropods as a whole. No mitochondrial genome from a pseudoscorpion has previously been published, so patterns of molecular evolution in pseudoscorpion mitochondrial genomes are completely unknown. Given the unusual patterns of molecular evolution found in other chelicerate mitochondrial genomes, we sought to determine whether pseudoscorpion mitochondrial genomes shared any of these unusual properties.

### Mitochondrial RNA structures and evolution

Transfer RNAs function to add peptides to an amino acid chain as they pass through the ribosome, and hence are essential for protein synthesis. Their cloverleaf-shaped secondary structure is highly conserved across all domains of life, which suggests that little structural deviation is tolerated for this essential molecule. In general, while metazoan mitochondria encode smaller tRNAs than their nuclear counterparts, they maintain sequence to encode a cloverleaf secondary structure. However, some metazoans show inferred structural variation in tRNAs encoded by the mitochondrial genome.

Metazoan mitochondrial genomes typically encode 22 tRNA genes, which are specific for the 20 essential amino acids, with alternative versions of tRNA-Ser and tRNA-Leu that possess alternate anticodons. Most metazoan mitochondrial genes for tRNA-Ser(AGN) lack the sequence to encode the DHU arm (also known as the dihydrouridine or D-arm). Because metazoans share this feature, it has been inferred that the loss of the D-arm from the *tRNA-Ser(AGN) *gene occurred before the divergence of metazoans [2]. Some diverse metazoan lineages possess a few mitochondrial tRNA genes that lack the canonical cloverleaf structure, and instead are missing either the TΨC arm (also called the pseudouridine or T-arm) or the D-arm. These few lineages are widely diverged, such that the gene reduction must be independently evolved. The first discovery of aberrant mitochondrial tRNA genes was from secernentean nematodes, in which all 22 mitochondrial tRNA genes lack sequence to encode cloverleaf tRNAs [3,4].

Some lineages of chelicerates have been found to possess transfer RNA genes that code for tRNAs whose secondary structures are inferred to lack one arm of the canonical cloverleaf tRNA. However, unlike in secernentean nematodes, chelicerates show variation among lineages as to whether a specific tRNA lacks an arm, and as to whether the missing arm is a T-arm or a D-arm [5]. Such aberrant tRNA genes have been found in spiders, acariform mites, vinegaroons, and scorpions (e.g. [5-10]). Opisthothelae spiders possess the most severely truncated tRNA genes yet found, and lack sequence to encode both a T-arm and the 3' aminoacyl acceptor stem [5,6,9]. In contrast, the mitochondrial genomes that have been sequenced so far from parasitiform ticks and mites, mesothele spiders, amblypygids, sea spiders, horseshoe crabs, ricinuleids, solifuges, and harvestmen all contain typical tRNA genes.

Mitochondrial genomes encode two ribosomal RNA subunits that form the core of the mitochondrial ribosome complex. Within arthropods, there is great variation in the reported length of these sequences, with most variation occurring in the length of the large rRNA subunit gene, often termed LSU rRNA, l-rRNA, or 16S. In chelicerates, the reported lengths of the LSU rRNA genes range from about 1000 to 1300 nucleotides (nts). The small ribosomal RNA gene, often termed SSU rRNA, s-rRNA, or 12S, also varies in size (from about 650 to 800 nts), but proportionally less so than the LSU rRNA gene.

Although reported lengths of the mitochondrial ribosomal genes vary greatly, it is not possible to accurately infer the lengths of these genes without knowing the secondary structures that they encode. This is because it is difficult to distinguish between intergenic sequence and the actual ends of the ribosomal genes. Hence, most reported lengths of the ribosomal genes actually report the length of the sequence between the genes that bound the ribosomal genes. It is difficult to infer secondary structure without having a good structural model for comparison. This task has been greatly aided by the publication of the crystal structure of a bacterial ribosome [11]. This study also mapped onto the rRNA secondary structure the points where the ribosomal RNA makes contact with the tRNA as it passes through the ribosome [11]. These contact sites are near the center of the ribosome, and due to their essential nature, should be conserved in all organisms.

### Gene rearrangements

The arrangement of the 13 protein-coding genes found in mitochondrial genomes is broadly conserved among the major animal phyla (see review by Boore [12]). Within Chelicerata, the same mitochondrial gene arrangement is shared by taxa from at least five orders, including Xiphosura, Amblypygi, and some taxa from Araneae, Parasitiformes, and Scorpiones [13,14]. This gene arrangement has been inferred to be the ancestral arrangement, because it is shared among these diverse lineages. This arrangement consists of 9 protein-coding genes and 13 tRNA genes encoded on the same strand. Because the majority of genes are encoded on this strand, it is referred to as the major strand, whereas the opposite strand is referred to as the minor strand.

In metazoans, tRNA genes are generally much more evolutionarily mobile than protein-coding genes [15], and among chelicerate orders, they show varying degrees of rearrangement. Taxa from the chelicerate orders Solifugae, Thelyphonida, and Ricinulei -- as well as Buthidae scorpions, Phalangiidae harvestmen, and opisthothele spiders -- differ in the location of some of their tRNA genes, while sharing identical protein-coding gene order. In chelicerates, rearrangements of mitochondrial protein-coding genes have been reported only for some parasitiform ticks [16] and a mite [17], some sea spiders [18,19], and all acariform mites [10,20,21].

### Nucleotide bias

Most metazoan mitochondrial genomes possess an overall cytosine and adenine (CA) nucleotide bias on their major coding strand [22]. This CA bias is thought to result from the asymmetrical way in which mitochondrial genomes replicate. During mitochondrial DNA replication in vertebrates, for example, the leading strand and lagging strand remain single-stranded for differing periods of time, leaving one strand more prone to mutations. This creates a mutational bias in the occurrence of deaminations of cytosine and adenine [23-25]. Despite strand specific mutational biases, metazoan lineages have been found to vary in their nucleotide bias, such that some taxa possess mt genomes with a guanine and thymine (GT) bias on their major strand. In arthropods, this reversed bias has been suggested to result from an inversion of the origin of replication [26], such that the opposite strand is now prone to deamination-induced mutational bias. If this is the case, then we would expect that changes in nucleotide skew would be correlated with genome rearrangements that affect the orientation of the origin of replication.

### Pseudoscorpion relationships

In order to interpret the evolution of genomic features, it is necessary to know the phylogenetic relationships of the organisms whose genomes have been sequenced. Among chelicerate lineages, some groupings have been recovered rather consistently in phylogenetic analyses, but many relationships have not been firmly established. The relationship of pseudoscorpions to the other chelicerates has not been well-established.

The chelicerate order Pseudoscorpiones contains 25 families and almost 3400 described species [27]. Some morphological analyses place Pseudoscorpiones as sister group to the order Solifugae [28,29], as do some analyses of morphology combined with molecular sequence data [30,31], although with low bootstrap support. Other combined analyses of morphology and molecular data place pseudoscorpions as sister to scorpions [32]. In both phylogenomic analyses of many nuclear loci [33] and analyses of ribosomal DNA sequences [32], the position of pseudoscorpions is essentially unresolved, with very low support. In sum, there is conflict among different types of data and in some cases very low support for the phylogenetic affinities of pseudoscorpions. This lack of consensus warrants the exploration of new data sources for phylogenetic analyses.

In this study, we sequenced the mitochondrial genomes of two widely divergent taxa of pseudoscorpions. We explore patterns of molecular evolution of the genome, as well as chelicerate relationships inferred by mitogenomic data. We find that all of the mitochondrial RNA encoding genes are greatly reduced in size in pseudoscorpions, and that this reduction in size is accompanied by loss of helices from the tRNA and LSU rRNA secondary structures. We examine patterns of mitochondrial genome nucleotide skew for all chelicerates in a phylogenetic framework.

## Results and discussion

### Genome size and arrangement

**Figure 1:**
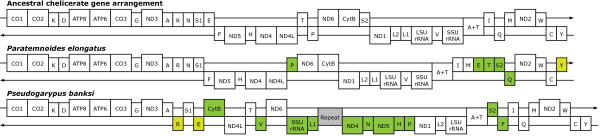
**The mitochondrial gene arrangements of the pseudoscorpions *Paratemnoides elongatus, Pseudogarypus banksi*, and the ancestral chelicerate**. The circular mt genome is depicted as linearized at the 5' end of the *CO1 *gene. The one letter amino acid code is used to designate the tRNA genes, with L1 = CUN; L2 = UUR; S1 = AGN; S2 = UCN. A + T is used to designate the putative control region. Genes above the medial line are encoded on the major strand, while those below the line are encoded on the minor strand. The green shading indicates genes that are in a different location than the ancestral gene arrangement, while the yellow shading indicates genes that are in the same location, but opposite strand, from the ancestral arrangement. Grey shading indicates a repetitive sequence insertion.

Both pseudoscorpion mitochondrial genomes are circular and encode the typical set of 37 mitochondrial genes. The mt genome arrangements of both pseudoscorpions differ from the ancestral chelicerate genome arrangement (Figure 1). The mt genome of *Pseudogarypus banksi *(Feaelloidea; Pseudogarypidae) [GenBank accession number: JQ040544] differs in the location of three of its 13 protein-coding genes (*ND4, ND5, Cytb*) and the SSU rRNA gene, relative to the ancestral arrangement. Additionally, 9 of the tRNA genes are in different locations (tRNAs specific for Arg, Glu, Val, Leu(CUN), Gln, His, Pro, Ser(UCN), and Phe). Of these tRNA genes, those coding for tRNA-Arg, tRNA-Glu, and tRNA-Gln are on the opposite strand as that in the ancestral condition. We also found a large region (~2650 nts) of repeated sequence located between the *tRNA-Leu(CUN) *and *ND4 *genes. Much of this region appears to consist of repeat units similar to the *tRNA-Lys *gene, although this gene is not located in the adjacent region. This duplicated region is responsible for a substantial increase in genome size compared with other chelicerates, which typically have genomes close to 15 Kb. The largest chelicerate mt genomes have been found in acariform mites in the family Trombiculidae (the largest mt genome is from *Leptotrombidium pallidum *at 16,779 nt) [34], and the genome of *Pseudogarypus *approaches this size at 16,546 nts.

In contrast, the size of the mt genome of *Paratemnoides *(Cheliferoidea; Atemnidae) [GenBank accession number: JQ040543] is 14,368 nts in length -- similar to the sizes of spider and mite mt genomes, but about 2200 nt shorter than that of *Pseudogarypus*. The mt genome of *Paratemnoides *is rearranged only with respect to six tRNA genes (tRNAs specific for Glu, Thr, Pro, Ser(UCN), Gln or Met, and Tyr). Of these, the genes coding for tRNA-Pro and tRNA-Tyr are located on the strand opposite of that of the ancestral condition.

None of the gene boundaries or inversions of genes onto the opposite strand that differ from the ancestral arrangement are shared between these two species (see Figure 1). This indicates that translocation of the genes must have occurred independently in these lineages, after their divergence from an ancestral pseudoscorpion.

The typical model to explain how mitochondrial gene rearrangements occur in animals is via tandem duplication of a part of the genome followed by random deletion of genes [35]. However, tandem duplication followed by random deletion cannot explain how genes come to be encoded on the opposite strand of a mitochondrial genome. Shao et al. hypothesized that inter-mitochondrial DNA recombination may explain how genes of chigger mites came to be encoded on the opposite mitochondrial strand [8,34]. Likewise, excision of a piece of the mitochondrial genome, followed by circularization, breakage of the circle, then recombination back into the original mitochondrial genome has been suggested to explain the current gene arrangement for the harvestman *Phalangium opilio *[14]. Recombination could also explain the current gene arrangements found in both of the pseudoscorpion mt genomes in the present study. Although recombination has not been thought to be important in the evolution of vertebrate mitochondrial genomes, it appears that it must occur among at least some chelicerate lineages.

Most chelicerate mitochondrial genomes maintain the same arrangement of protein-coding genes [13,14]. However, all mitochondrial genomes of acariform mites possess rearranged genes, many with rearranged protein-coding genes [21]. The pseudoscorpion *Pseudogarypus *also possesses rearranged protein-coding genes. It shares this feature with acariform mites, but not with all pseudoscorpions, as *Paratemnoides *has the same protein-coding gene order as the ancestral chelicerate.

### tRNA genes

**Figure 2:**
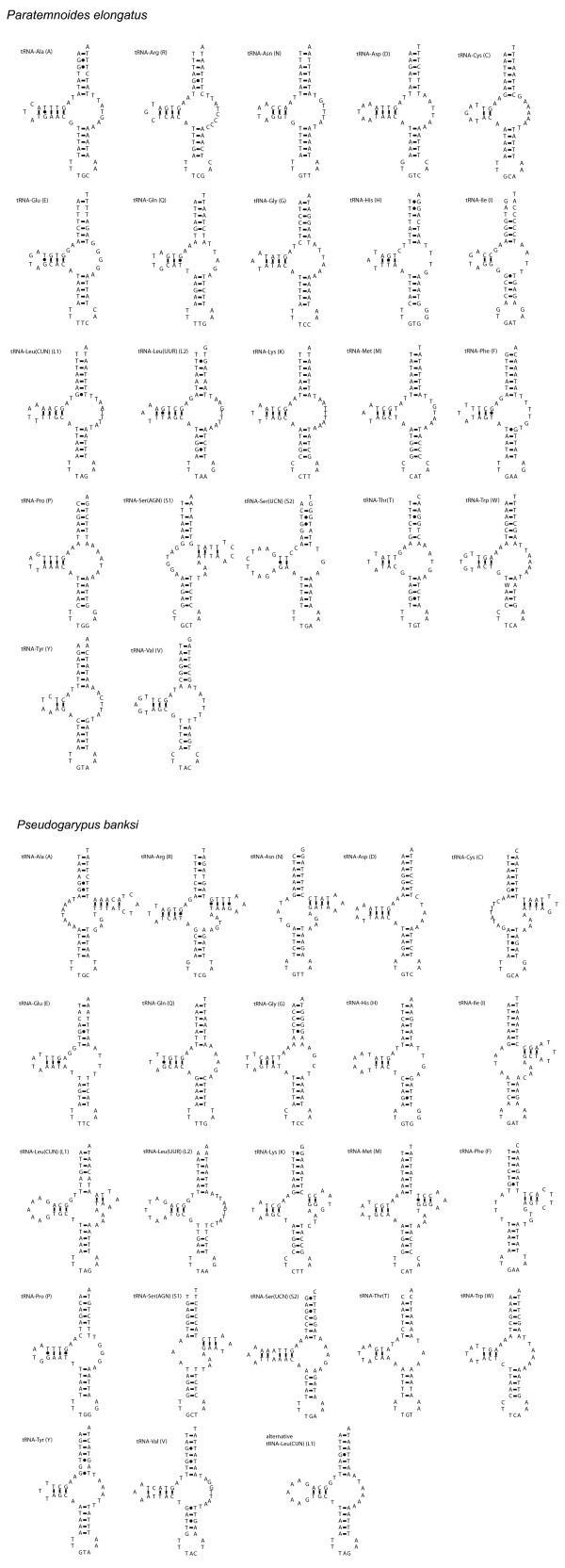
**The inferred secondary structures of the 22 mitochondrial tRNAs**. Structures are arranged in alphabetic order of the amino acids they recognize. The standard three-letter abbreviation for the amino acid the tRNA recognizes is given, and in parentheses the one letter amino acid abbreviation is given. A dash indicates a Watson-Crick bond, and a dot between G and T indicates a bond between G and uracil. A) *Paratemnoides elongatus *tRNA structures. B) *Pseudogarypus banksi *tRNA structures. Two alternative structures are shown for tRNA-Leu(CUN), as described in the text.

All mt tRNA genes of *Paratemnoides *lack sequence to encode a cloverleaf tRNA secondary structure. Instead, 21 of these genes are inferred to encode tRNAs that lack a T-arm (Figure 2a), while the gene coding for tRNA-Ser(AGN) lacks the sequence to encode a D-arm.

In *Pseudogarypus*, only 3 or 4 of the mt tRNA genes have the potential to form a cloverleaf tRNA. It is possible that the *tRNA-Leu(CUN) *gene encodes a tRNA with a cloverleaf secondary structure, but if so, there are two mismatches in the acceptor stem, and a weak 2-bp T-arm stem. Alternatively, the *tRNA-Leu(CUN) *gene encodes a tRNA that lacks a T-arm, but has a perfectly paired acceptor stem. Both alternatives are illustrated in Figure 2b. If tRNA-Leu(CUN) lacks a T-arm, then a total of 13 tRNA genes are inferred to encode tRNAs that lack T-arms. The other 6 mt tRNA genes are inferred to encode tRNAs that lack D-arms, including the gene coding for tRNA-Ser(AGN) (Figure 2b).

Although some arachnid lineages contain species whose mitochondria possess many tRNA genes without sequence to encode a cloverleaf-shaped tRNA, the pseudoscorpions in this study show some of the most widespread losses of tRNA arms yet documented among metazoans. The only other chelicerate lineages to show this extent of loss from their tRNA genes are acariform mites. Some acariform mites have been found to possess mitochondrial genomes in which all 22 of their tRNA genes are inferred to encode tRNAs that lack either a D-arm or a T-arm [10,21,36]. *Paratemnoides *joins the short list of taxa with mt genome-wide losses of canonical tRNA genes. In addition to some chelicerates and secernentean nematodes, gall midge insects have also been found to have severely truncated tRNA genes [37], similar to those of opisthothele spiders. So far, genome-wide losses of T- or D-arm-encoding mitochondrial tRNA sequences have been restricted to the Ecdysozoa.

Within spiders, there is variation among taxa in the identity and number of tRNA genes that lack T-arms. When this variation was traced onto a phylogenetic tree, it was found that once a tRNA gene loses sequence to encode a D- or T-arm, sequence to encode these arms is not regained [5]. We find variation among these two pseudoscorpion lineages in whether the D- or T-arm sequence has been lost from a given tRNA gene. These two pseudoscorpions show shared losses of T-arms from 12 or 13 tRNA genes that are specific for Asp, Glu, Gln, Gly, His, Leu(UUR), Pro, Ser(UCN), Thr, Trp, Tyr, Val, and likely Leu(CUN). However, in *Pseudogarypus *the tRNA genes specific for Ala, Asn, Cys, Ile, and Phe all lack D-arms. The loss of D-arm sequence from these 5 tRNA genes must have occurred after the divergence of the major pseudoscorpion lineages, because the tRNA homologues in *Paratemnoides *possess D-arm sequence, but lack T-arm sequence. The *tRNA-Arg, tRNA*-Lys, *tRNA-Met*, and perhaps the *tRNA-Leu(CUN) *genes, code for cloverleaf-shaped tRNAs in *Pseudogarypus*, therefore we can infer that the ancestral pseudoscorpion also possessed full-length tRNA genes for these tRNAs. In sum, based upon these two genomes from the deeply diverged pseudoscorpion lineages Feaelloidea and Cheliferoidea, we can infer that the common ancestor of extant pseudoscorpions had likely lost sequences to encode canonical secondary structures for multiple different mt tRNAs.

It is not clear why some lineages of chelicerates have lost sequence to encode either the D-arm or T-arm from their mt tRNA genes. In general, chelicerates that lack canonical mt tRNA genes also tend to have rearranged mt genomes. For example, all opisthothele spiders and acariform mites have mt tRNA genes that are rearranged with respect to the ancestral chelicerate, and all possess many mt tRNA genes that lack the ability to encode cloverleaf tRNAs. However, while this trend is also true within pseudoscorpions, it does not explain the variation in tRNA gene reduction. The more rearranged mt genome of *Pseudogarypus *has fewer non-canonical tRNA genes (19 out of 22), whereas *Paratemnoides *exhibits less rearrangement, yet all its tRNA genes lack sequence to encode cloverleaf tRNAs.

### rRNA genes

The ribosomal RNA genes of both pseudoscorpions are extremely short for chelicerates and other arthropods. *Pseudogarypus *has a smaller mt LSU rRNA than *Paratemnoides *(about 986 nts versus 1013 nts). This is one of the shortest mt LSU rRNA lengths reported for any chelicerate; only some acariform mites and some spiders possess equally short LSU rRNA genes. Typically, chelicerates have LSU rRNA genes of 1200 to 1300 nts. The three orders of chelicerates that have historically been inferred as most closely related to pseudoscorpions (based on morphology) are harvestmen, solifuges, and scorpions. The mitochondrial genomes of representative taxa from these lineages have LSU rRNA genes ranging from about 1150 nts (Scorpiones: *Buthus occitanus*) to 1250 nts (Opiliones: *Phalangium opilio*; Solifugae: *Eremobates palpisetulosus *group).

**Figure 3:**
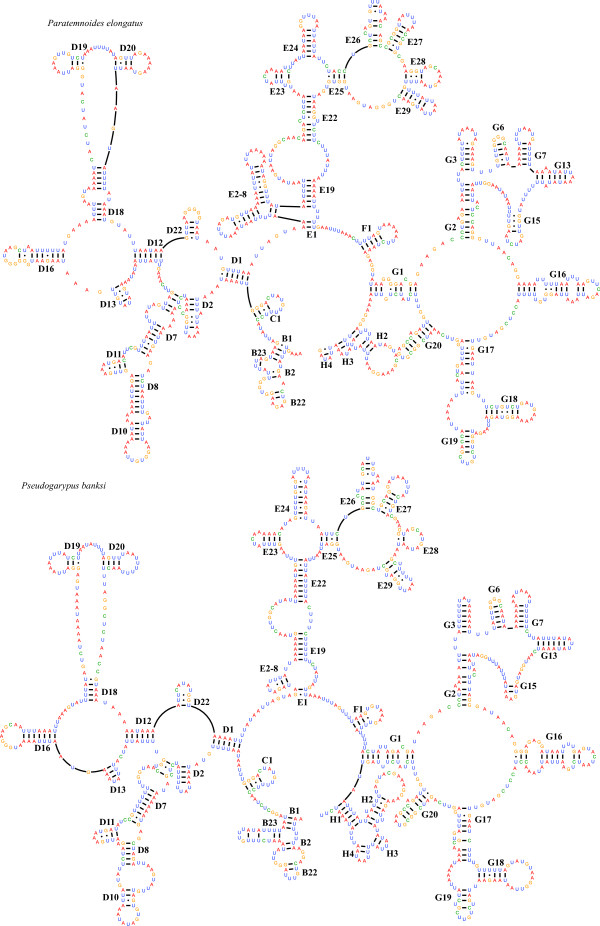
**Inferred secondary structures of the mitochondrial large subunit ribosomal RNA**. The numbering of the helices follows Wuyts et al. [39]. Each nucleotide is color coded, where red = adenine, blue = uracil, green = cytosine, and yellow = guanine. A dash indicates a Watson-Crick bond, and a dot between G and U indicates a nonWatson-Crick bond. A) *Paratemnoides elongatus *LSU rRNA B) *Pseudogarypus banksi *LSU rRNA.

Decreases in size of the LSU rRNA genes could be due to reductions spread throughout the gene, or reductions concentrated in specific areas, such as the ends of the gene. Reductions that occurred throughout the gene would result in decreases of the sizes of many to most of the helices in the secondary and tertiary structures of the rRNA. Reductions concentrated in specific areas could cause losses of entire helices, similar to the inferred losses of helices that have occurred in pseudoscorpion mt tRNAs. We inferred the secondary structures of both pseudoscorpion LSU rRNAs, to determine where within the molecule the reductions had occurred. These structures are depicted in Figures 3A and 3B. To assess the location and extent of reductions, we compared the secondary structures we inferred for the LSU rRNA genes from both pseudoscorpions to that of the opilionid *Phalangium opilio *[14]. We found that both of the pseudoscorpions have lost helices D14-D15 (also referred to as helix 38 of domain II in the bacterial LSU rRNA), which should be located between helices D13 and D16 (Figures 3A and 3B). This two-part D14-D15 helix is typically present in the arthropod LSU rRNAs secondary structures that have been inferred [38]. Within chelicerates, helices D14-D15 are present in the amblypygid *Damon diadema *[39] and in the harvestman *Phalangium opilio *[14]. These helices are missing in some of the acariform mites that have been analyzed (e.g. *Panonychus citri *[10]), but appear to be present in other Acariformes (e.g. *Dermatophagoides pteronyssinus *[21] and *Leptotrombidium *[8]).

The crystal structure of the bacterial ribosome of *Thermus thermophilus *has been used to infer the contact sites between the RNA core of the ribosome and tRNAs during translation [11]. Based on our comparison of inferred LSU rRNA secondary structures of pseudoscorpions to the structure of the LSU rRNA of *T. thermophilus*, the nucleotides at the end of helix D15 should make contact with the D and T loops of the A-site tRNA within the ribosome during translation. The D14-D15 helices are lacking in both pseudoscorpion LSU rRNAs, suggesting either that this contact point has been lost and is not essential for protein synthesis, or that this tRNA-ribosome contact site has moved to another location.

The G4 helix of the LSU rRNA is also not present in either pseudoscorpion (Figures 3A and 3B). This helix should be encoded immediately downstream of the G3 helix, but instead, only a short G3 helix is present. The G4 helix (referred to as helix 77 of domain V in the bacterial LSU rRNA) is located at the core of the bacterial ribosome, and makes contact with the D and T loops of the E-site tRNA as the tRNA exits the ribosome during translation [11].

Both pseudoscorpions lack a large region of the 5'end of the LSU rRNA gene, which typically codes for a series of helices, referred to as domain I in the bacterial LSU rRNA, or following the notation in [40], the B region. The secondary structure for this region was difficult to infer accurately, so we have less confidence in the accuracy of our reconstruction. However, it appears that helices B3, B10, and B12 have all been lost relative to those of *Phalangium*. Nucleotides in the B10 helix (or helix ll, domain I in the bacterial LSU rRNA) base pair with the E-site tRNA as the tRNA exits the ribosome [11].

Two other regions of helices have been lost in one or both pseudoscorpions LSU rRNAs, but all of the other tRNA-rRNA contact sites appear to be present in both pseudoscorpions. Both pseudoscorpions lack the D23 helix, which should be located immediately adjacent to the D22 helix, or it may instead be very reduced compared to that of *Phalangium*. The LSU rRNA of *Pseudogarypus *lacks helices E1-E8 that are present in *Paratemnoides*. This helix is present in harvestmen [14], and in amblypygids [39].

In summary, we find that the reduction in length of the *LSU rRNA *gene was accompanied by the loss of some helices from the secondary structure of the LSU rRNA. Both pseudoscorpions share the losses of the same 4 helices, at least two of which appear to be RNA-RNA contact points within the ribosome. We infer that the loss of these helices must have occurred after the divergence of the pseudoscorpion ancestor from harvestmen, as *Phalangium opilio *possesses these helices.

The lengths of the small ribosomal RNA genes are also reduced in pseudoscorpions, although proportionally less than the LSU rRNA genes. The absolute length of these genes in chelicerates can be more difficult to determine than that of the *LSU rRNA *gene, because the 5' end of the *SSU rRNA *gene typically abuts the non-coding region (although this is not the case in *Pseudogarypus*), which is poorly conserved in chelicerates. Additionally, while the 5' end of the SSU rRNA is structurally fairly conserved, its sequence is not conserved. Therefore, only approximations of gene length are possible when we have only DNA sequence data as a guide. *Pseudogarypus *has a smaller mt SSU rRNA than *Paratemnoides *(about 687 nts versus 727 nts). These are somewhat shorter lengths than the mt *SSU rRNA *genes of harvestmen, solifuges, and scorpions. The mt *SSU rRNA *genes in those taxa range from 725 nts in solifuges (*Eremobates palpisetulosus *group) to 768 nts in harvestmen (*Phalangium opilio*) to about 790 nts in scorpions (*Uroctonus mordax *and *Buthus occitanus*).

During protein synthesis, the SSU rRNA region of the ribosome makes contact primarily with the anticodon region of the tRNA, while the LSU rRNA has contact sites with the T-arm and with the D-arm of the tRNA (for specific details, see [11]). Therefore, if the tRNAs of pseudoscorpions are structurally coevolving with the ribosome that they interact with, we may predict that we would see length differences primarily in the LSU of the ribosome, but not necessarily in the SSU of the ribosome. Additionally, we would predict that the length differences would primarily be concentrated in the rRNA-tRNA contact sites of the LSU of the ribosome.

It has previously been shown that the substitution rate for the nucleotide sites in the *LSU rRNA *gene scale with their distance from the center of the ribosome, across all domains of life [40]. This finding corroborates how important the core of the ribosome is for all organisms. The loss of tRNA-ribosome contact sites in pseudoscorpion LSU rRNA suggests that pseudoscorpion mt ribosomes may have evolved some remarkable changes in their structure and interactions with their tRNAs.

We have previously suggested that shortened ribosomes are correlated with shortened tRNA genes in chelicerates [6,41]. The finding of shortened tRNA and rRNA genes in pseudoscorpions provides an additional example of such a co-occurrence. The co-occurrence of short RNA-encoding genes may be due to pressures to decrease genome size overall, or due to compensatory evolution of the tRNAs with the ribosome with which they interact. If tRNA-ribosome contact sites were lost or mutated in the ribosome, it would have allowed the tRNA genes to accrue mutations at these contact sites. Non-functional sites could be deleted from the tRNA genes via random genetic drift. Alternatively, if there is a replicative advantage to having a small genome, then selection could act to eliminate non-essential regions of the genome.

However, the compact tRNA and LSU rRNA genes of *Pseudogarypus *are not mirrored by a compact genome. Its mt genome possesses an apparently non-coding region that increases the size of the genome substantially over that of *Paratemnoides*. It is plausible that this non-coding region is due to a relatively recent insertion, and that selection for small genome size has not occurred long enough for us to see the evolutionary reduction in, or loss of, this insert. Alternatively, it is possible that the insert is more ancient, and that selection has acted to a greater degree to eliminate RNA helices in the ribosome and tRNAs in a concerted manner. Our findings of losses of tRNA-rRNA contact sites in both the tRNAs and the LSU rRNA suggest that natural selection has acted such that these structures have coevolved to maintain function during protein synthesis.

### Phylogenetic analyses

The data set used for phylogenetic analyses consisted of 2907 amino acids, with about 73% of the initial alignment of 3975 amino acids retained after Gblocks trimming of the 13 protein-coding alignments. The level of sequence conservation varied among genes. The most conserved genes retained over 90% of their amino acids (*CO1, CO3*, and *Cytb*), many genes retained 60-76% of their amino acids (*ATP6, CO2, ND1, ND3, ND4*, and *ND5*), and the *ATP8, ND2, ND4L*, and *ND6 *genes possessed the lowest levels of sequence conservation (44-54% amino acids retained).

**Figure 4:**
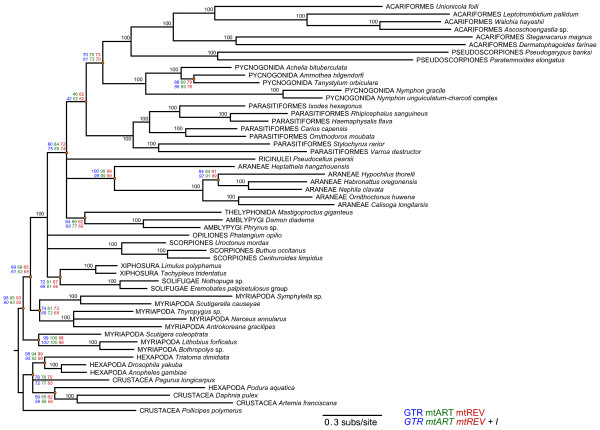
**Consensus tree of 6 different maximum likelihood analyses of amino acid data using different models of evolution**. All analyses used the gamma correction model for rate heterogeneity. Bootstrap support values for the 6 analyses are given next to each node. The bootstrap values for the 6 different analyses using 6 models of evolution are given in the following order: Top line = GTR (colored blue); mtART (colored green); mtREV (colored red); Bottom line = GTR + I (colored blue); mtART + I (colored green); mtREV + I (colored red). When all 6 analyses had the same bootstrap value, only one value is given (colored black). Nodes with bootstrap support of less than 60% were manually collapsed. See text for further details of analyses.

Phylogenetic analyses of all 13 mitochondrial protein-coding gene sequences from a diverse group of chelicerates (see Additional file 1 for a taxonomic summary) yield phylogenetic trees that consistently recover the pseudoscorpions as a monophyletic group (Figure 4). Surprisingly, pseudoscorpions are found as sister group to acariform mites, with 100% bootstrap support. The arachnid order Acari is found to be paraphyletic, with one well-supported clade of Acariformes and one clade of Parasitiformes. This result is in agreement with other recent analyses that have recovered Acari as diphyletic [21,32,42]. We evaluated the alternative hypothesis, that Acari is a monophyletic group, but this hypothesis was rejected by the Approximately Unbiased (AU) test (*P *= 0.0005), with a Ln likelihood score of -182198.24 for the unconstrained tree, and a Ln likelihood score of -182247.47 for the monophyletic Acari topology. The other orders for which we have data from multiple mitochondrial genomes also are recovered as monophyletic: the spiders (Araneae), scorpions (Scorpiones), sea spiders (Pycnognida), and ticks and parasitic mites (Parasitiformes). Hence, except for Acari, our analyses always recover all named orders of arachnids as monophyletic. Our analyses also always recover the subphylum Chelicerata with high support. In contrast, we do not find Arachnida to be monophyletic, due to the placement of the Xiphosurans (horseshoe crabs) and Pycnogonida (sea spiders) amongst the arachnids. We examined the alternative hypothesis of arachnid monophyly, but this hypothesis was rejected by the AU test (*P *= 0.001; Ln likelihood of -182338.12 for the monophyletic Arachnida tree). We also did not find support for Pseudoscorpiones as the sister lineage to Solifugae, as the AU test strongly rejected this hypothesis (*P *< < 0.001; Ln likelihood score of -182358.64).

We found that the protein-coding genes of both pseudoscorpions and acariform mites are characterized by high numbers of substitutions, resulting in long branches in phylogenetic analyses. Additionally, we know that variation in amino acid skew is found among chelicerates [43]. Therefore, we undertook a series of analyses to determine whether the placement of these two lineages as sister groups could be caused by an artificial grouping due to saturation of the sequences or due to similarities in amino acid skew.

To determine whether long-branch-attraction due to sequence saturation could be causing Acariformes to group with Pseudoscorpiones, we first eliminated acariform taxa with particularly long branches from our analyses. We found that this had no effect on the placement of pseudoscorpions as sister to acariform mites (trees not shown). In addition to likelihood analyses, we also undertook a series of Bayesian analyses, using the same models of evolution as for likelihood. All Bayesian analyses recovered Acariformes as the sister clade to Pseudoscorpiones with high posterior probabilities.

We also recovered the sister-group relationship of Acariformes with Pseudoscorpiones in all 34 chains we ran with Phylobayes using a CAT site-heterogeneous mixture model. In 18 of these chains, pycnogonids were recovered as the sister group to other chelicerates, whereas in 16 analyses they placed as derived within the arachnids. In no analysis did we recover a monophyletic Arachnida, as horseshoe crabs always were recovered as the sister lineage to solifuges (trees not shown).

Coding mitochondrial nucleotides as either purines or pyrimidines (RY recoding) has been shown to recover deep-level relationships and to increase phylogenetic signal [44,45]. We implemented RY recoding at 3^rd ^codon positions only and at 1^st ^and 3^rd ^codon positions, and in both cases we recovered Acariformes as the sister clade to Pseudoscorpiones with 92% and 70% bootstrap support, respectively (trees not shown). We found 62% bootstrap support for this grouping when we implemented a variation of RY recoding termed the Neutral Transitions Excluded (NTE) method [46].

It was striking that both Acariformes and Pseudoscorpiones show long branches on the amino acid phylogenetic trees. This indicates that a large number of amino acid substitutions have occurred within these lineages. To reduce the possibility that amino acids are saturated and causing long-branch-attraction artifacts, we recoded amino acids into 6 physiochemical functional groups. We have previously found this method successful at recovering some nodes among chelicerate lineages [43]. The resultant phylogenetic tree (not shown) still recovered Acariformes and Pseudoscorpiones as sister lineages with 100% bootstrap support.

The mitochondrial genome comprises only a portion of an organism's overall genetic makeup, and some researchers advocate using a combination of both organelle and nuclear loci when making phylogenetic inferences (e.g. [47]). When using only a region of an organism's genetic makeup to infer its evolutionary history, several potential problems could exist. The genetic marker may show a pattern of retention of an ancestral polymorphism if it is used to infer recent divergences, and therefore may not accurately reflect species divergence. However, incomplete sorting of ancestral polymorphisms should not be a problem when making phylogenetic inferences among ancient lineages, such as among chelicerates. Genetic markers for inferring ancient divergences may be problematic if the sequences are so diverged that they are saturated, or alternatively, if mutational bias or natural selection has influenced sequence composition. If any of these occur, it may lead to incongruence among different data sets, and to incorrect phylogenetic hypotheses. Although mtDNA has generally been viewed as evolving neutrally, and therefore not likely to cause incorrect phylogenetic inferences due to selection, pronounced convergent selection has been found to influence the evolution of some mitochondrial genomes [48].

### Base composition and nucleotide bias

**Figure 5:**
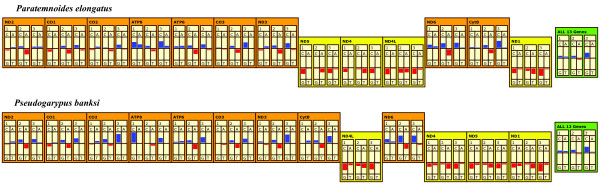
**The nucleotide skew for each of the 13 mitochondrial protein-coding genes of *Paratemnoides *and *Pseudogarypus***. The genes are arranged in the order they are located in the mitochondrial genome, with the circular genome arbitrarily linearized at the 5' end of the *ND2 *gene. Boxes shaded orange and offset upwards depict genes encoded on the major strand, while boxes shaded yellow and offset downward depict genes encoded on the opposite strand. The green shaded box depicts the skew averaged across all 13 genes. For each gene, the average AT and CG skews at each codon position are shown. Blue depicts cytosine and adenine skew, red depicts guanine and thymine skew. An excess of cytosine is depicted as an upward blue bar, while an excess of guanine is depicted as a downward red bar. An excess of adenine is depicted as an upward blue bar, while an excess of thymine is depicted as a downward red bar.

The nucleotide composition of both pseudoscorpion mt genomes shows an overall AT bias. The mt genome composition of the major coding strand of *Pseudogarypus *possesses an AT frequency of 76.9%, while that of *Paratemnoides *is 73.8%. Both mt genomes also show CG nucleotide skews on their major coding strands. The overall CG skews for each of the major coding strands of the mitochondrial genomes are 0.23 for *Paratemnoides *and 0.28 for *Pseudogarypus*. This is almost identical to the CG skews for all 13 of the protein-coding genes, which showed a CG skew of 0.23 in *Paratemnoides*, and 0.27 for *Pseudogarypus*. The CG and AT skews of each of the protein-coding genes, linearly arranged along the lengths of their genomes, is graphically depicted in Figure 5. Third-codon positions tend to show a greater skew than either first or second positions (Figure 5), with an average CG skew of 0.46 for *Paratemnoides *and 0.55 for *Pseudogarypus*. However, there is gene-by-gene variation in CG skew for each codon position (Figure 5 and Additional file 2).

### Distribution and evolution of nucleotide bias among chelicerates

A CA bias on the major strand of the mt genome is typical of many chelicerates, and of arthropods in general. The amount of variation in nucleotide skew that has been found within Chelicerata is among the greatest in any arthropod lineages. Some distantly related orders of arachnids -- such as spiders, scorpions, and some acariform mites -- possess mt genomes with a pronounced GT bias e.g. [21,43,49]. However, many chelicerate lineages -- including xiphosurans, amblypygids, vinegaroons, and camel spiders -- possess a CA nucleotide bias on their major coding strand [43]. Within the chelicerate group Pycnogonida (sea spiders), there exists wide variation in mt genome nucleotide skew, ranging from a pronounced GT bias to a CA bias [19].

**Figure 6:**
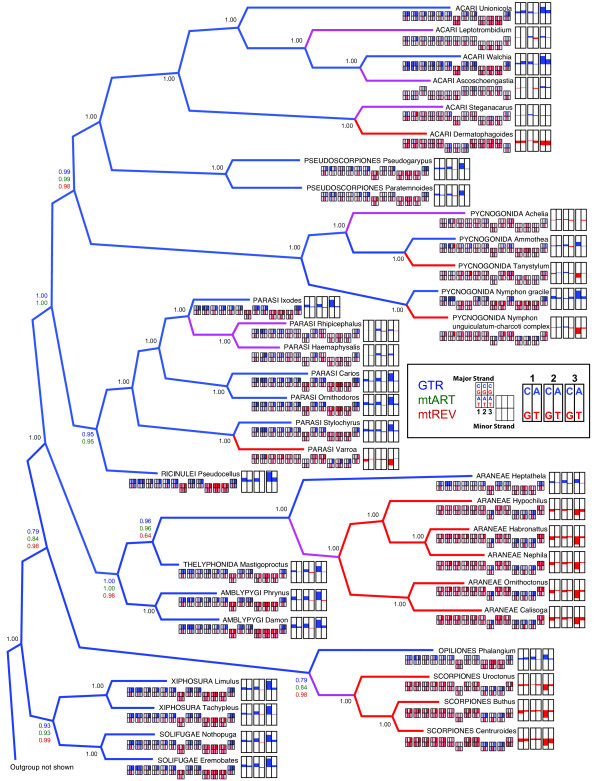
**Phylogenetic tree of chelicerates based on amino acid data, with nucleotide skew shown on the terminal nodes**. This Bayesian consensus tree was generated using GTR, mtART, and mtREV models of evolution for amino acids. Site-specific rates across sites were modeled with a Dirichlet process. Posterior probability values are next to the nodes, but when all 3 analyses had the same value, only one value is given (colored black). Outgroup taxa are not depicted. The branches are colored blue if the terminal taxa possess a positive skew towards C or A at 3^rd ^codon positions, red if they possess a negative skew (i.e. G or T skew), or purple if they lack a strong skew (i.e. skew between -0.2 and +0.2). For each terminal taxon, the AT and CG skew of each codon position of the 13 protein-coding genes is shown under the taxon name. The genes are arranged in alphabetical order (*ATP6, ATP8, CO1, CO2, CO3, Cytb, ND1, ND2, ND3, ND4, ND4L, ND5 *and *ND6*) to allow gene-by-gene comparisons among otherwise rearranged genomes. Genes located on the minor strand are distinguished by the boxes offset downwards. The skew of each codon position for each of the 13 genes is represented by a color gradient, in which the skew ranges from -1 (red) to 0 (white) to +1 (blue). At the terminal node is a summary of CG and AT skew for each of the codon positions for all 13 genes. Blue depicts cytosine and adenine skew, red depicts guanine and thymine skew. An excess of cytosine is depicted as an upward blue bar, while an excess of guanine is depicted as a downward red bar. An excess of adenine is depicted as an upward blue bar, while an excess of thymine is depicted as a downward red bar.

To better understand the distribution of nucleotide skew, we plotted its distribution onto a phylogenetic tree. To more fully comprehend the nuances in nucleotide skew among different protein-coding genes, we examined skew separately for each of the 13 genes, and at each of the three codon positions. Because this is an immense amount of information, we present a visual overview of the skew distribution for each codon position and for each gene in Figure 6. Because gene order varies among the different chelicerate taxa, the genes are arranged in alphabetical order, to allow gene-by-gene comparisons between taxa. Additional file 2 provides the data that was used to create these graphics, and the protein-coding gene arrangement found in these taxa.

We find that skew varies dramatically among chelicerate taxa. Some taxa show a clear CA bias (colored blue in Figure 6) for all of their protein-coding genes located on the major strand. These include the taxa *Walchia *and *Pseudocellus *from the divergent Acariformes and Ricinulei lineages. Other taxa have a strong GT bias (colored red in Figure 6) on their major coding strand, including all scorpions and all true (opisthothele) spiders. Most taxa, including both pseudoscorpions, exhibit a CA bias at their 1^st ^and 3^rd ^codon positions, but a CT bias at 2^nd ^positions. Some taxa show little bias in their nucleotide composition at 3^rd ^codon positions (branches colored purple in Figure 6).

Among many chelicerate lineages, there appears to be a seemingly random phylogenetic distribution of nucleotide skew. The Pycnogonida display everything ranging from a strong CA bias to a marked GT bias to an almost non-biased nucleotide distribution. This degree of variation exists even within a single genus, with one species of *Nymphon *possessing a strong CA bias and another a strong GT bias. Acariformes also show a range of skew values, from CA-biased to GT-biased to very little nucleotide bias.

In contrast, skew seems to be conserved in some chelicerate lineages. A GT bias apparently arose after the divergence of true spiders from their common ancestor with mesothele spiders, and the opisthothele lineage has remained GT-biased since then. Likewise, all scorpions sequenced so far exhibit a GT bias, suggesting that this trait was inherited from the common ancestor of scorpions, and retained throughout scorpion's evolutionary history.

These patterns of skew distribution do not seem to coincide with gene rearrangements (see Additional file 2). It has been suggested that inversions of the origin of replication could have caused changes in mutational biases on the major strand from CA to GT in multiple arthropods [46,50]. If this is the case, then it may be expected that genomes prone to rearrangements are also the ones that tend to have major coding strands that are no longer CA-biased. Additionally, if changes in nucleotide skew on the major strand are due to inversion of the control region or origin of replication onto the minor strand, then recombination must have occurred. Given the high degree of variation in nucleotide skew in chelicerates, this would suggest that recombination has occurred fairly frequently within chelicerate mitochondrial genomes. It is not known with certainty where the origin of replication is located within chelicerate mitochondrial genomes, or even whether there are multiple origins. The origin of replication was determined for multiple species of insects, and found to differ in location between holometabolous and hemimetabolous insects [51]. Additional studies such as these are needed in chelicerates in order to understand how the origin of replication influences the nucleotide biases of the mt genome.

It has previously been found that bacteria show a mutational bias toward AT [52]. It is argued that variation in AT nucleotide content found among bacterial genomes is best explained either by selection acting on the probability of fixation of mutations, or by selection favoring biased gene conversion [52,53]. Mitochondria are derived from bacteria, so it may not be surprising that most mitochondrial genomes show a similar AT bias. Likewise, the variation in nucleotide content found among chelicerate mitochondrial genomes on the major coding strand either must be due to natural selection affecting which mutations becomes substituted, or must reflect neutral substitutions governed by mutational bias. Previous reports have assumed that the variation in nucleotide skew found among chelicerates is due to changes in the mutational bias within the mitochondrial genome [46]. However, it is also plausible that selection has helped to shape nucleotide use in genomes that do not have a CA skew. A phylogenetic pattern of seemingly random distribution of a trait is consistent with a pattern selection may leave if it has acted upon that trait. The evolutionary reversal of a CA bias to a GT bias found in some lineages of chelicerates may be due to selection, rather than to neutral processes, although it is not clear what the selective force may be.

### Phylogenetic implications

It is somewhat surprising that all our phylogenetic analyses of mt protein-coding genes are in agreement that pseudoscorpions and acariform mites are sister lineages. Previous analyses using nuclear-encoded genes have found either conflicting placements of pseudoscorpions depending upon the method of analysis, or less than 50% bootstrap support for the placement of pseudoscorpions [32,33]. While a plethora of different phylogenetic hypotheses for the relationships of pseudoscorpions have been proposed by previous workers, morphological hypotheses have tended to place pseudoscorpions as most closely allied to solifuges, scorpions, and harvestmen. No phylogenetic hypothesis proposed thus far has had a high level of assessable, statistical support. However, a quandary exists in how to interpret our phylogenetic data, as our results would require independent origins of some morphological traits that may be expected not exhibit homoplasy.

The structure of sperm and male genitalia suggests that Pseudoscorpiones are not closely related to Solifugae [54], but instead, the similarities of sperm structures in Solifugae suggest that they are the sister group to Acariformes [55]. Recent analyses of nuclear rRNA sequences also support a sister-group relationship of Solifugae with Acariformes [32]). These authors also discuss several morphological synapomorphies that Solifugae shares with Acariformes.

We do not recover such a grouping with our mitochondrial data, and instead repeatedly find strong support for an Acariformes + Pseudoscorpiones clade, as well as a distantly related Xiphosura + Solifugae clade. In light of the well-supported relationship between pseudoscorpions and Acariformes, we are led to ask whether any morphological characters might also support this surprising relationship. Both pseudoscorpions and mites possess trachea, but it is known that other chelicerate lineages, such as Opiliones, also possess trachea. In fact, there is some historical precedent for grouping the tracheate arachnids, but this grouping has been largely dismissed by arachnologists because trachea are known to have arisen independently within some arthropod lineages. Dunlop and Alberti [56] summarized the similarities of the parasitiform and acariform lineages with other arachnids. They describe two features that are shared among these lineages and pseudoscorpions. These are the presence of a ventrally moving finger on the chelicera, and the presence of an anterior projection termed the epistomal plate, which possesses flap-like lips. However, these features are also shared with Solifugae, a group that we recover as most closely related to horseshoe crabs.

There are features at the molecular level that are shared by Acariformes and Pseudoscorpiones. Intriguingly, the mt genomes of acariform mites also exhibit widespread loss of arms from their tRNA genes. In fact, no acariform mites or pseudoscorpions so far sequenced possess a complete set of "typical" tRNA genes, but instead some to all of their tRNA genes do not encode either D-arm or T-arm sequences. As discussed previously, these taxa vary as to which tRNAs are missing an arm, and whether it is a T-arm or D-arm. Therefore, it is not likely that acariform mites and pseudoscorpions share the loss of a specific tRNA helix as a synapomorphy. Instead, it is more likely that they share the propensity to lose helices from their tRNA genes.

Both pseudoscorpions and acariforms have extremely short ribosomal RNA genes. We have shown that losses of helices of specific regions of the LSU rRNA are shared by both pseudoscorpions, and therefore these losses likely pre-date the loss of sequence to encode cloverleaf shaped tRNAs. Further analyses of additional pseudoscorpion and acariform mite rRNA secondary structures will allow insight into whether these lineages share the loss of helices that may be involved in tRNA D- or T-loop contact with the ribosome.

Acariform mites and pseudoscorpions also exhibit extensive gene rearrangements. However, these taxa do not share the same gene arrangement, and even the two pseudoscorpions differ in their genome organization. To date, the only other chelicerates that have been found to have genome rearrangements of protein-coding genes are some Parasitiformes, and pycnogonids in the family Nymphonidae. Intriguingly, these orders were recovered as a monophyletic group in our phylogenetic analyses (Figures 4 and 6).

### Rate of molecular evolution

The pseudoscorpion amino acid sequences are on long branches on our phylogenetic trees. Because branch length is proportional to the number of substitutions, it indicates that many substitutions separate *Pseudogarypus *from *Paratemnoides*, and from their most recent common ancestor with other chelicerates. These branches are longer than those found among any other chelicerate lineage, except for Acariformes (see Figure 4). This indicates either that pseudoscorpions and Acariformes are more ancient lineages than other chelicerate lineages, or that they have elevated rates of sequence evolution. These two alternate scenarios are difficult to tease apart.

Fossil pseudoscorpions dated to about 380 million years old have been found in Devonian deposits [1]. Even older acariform mite, opilionid, and scorpion fossils have been found, and are dated to 410-428 mya (reviewed by Dunlop and Selden [57]). Therefore, fossil data alone does not allow us to infer that pseudoscorpions have accumulated more mutations than scorpions and spiders simply because they are older. The lack of an older pseudoscorpion fossil does not necessarily indicate that the lineage is not older, as it is possible that older fossils remain to be discovered.

Pseudoscorpion mt genomes show some gene rearrangements, including rearranged protein-coding genes in *Pseudogarypus*. Some studies have linked the degree of gene rearrangements with the rate of molecular evolution [58]. The degree of mitochondrial protein-coding gene rearrangements and the rate of evolution may indeed be somewhat interrelated in chelicerates. The acariform mites, pycnogonids in the family Nymphonidae, and pseudoscorpions all have protein-coding genes that are rearranged from the ancestral condition, and all show many amino acid substitutions, i.e. long branches, on the phylogenetic trees we reconstructed, consistent with elevated rates of substitution.

## Conclusions

We find that all of the RNA-encoding genes in pseudoscorpion mitochondrial genomes are greatly reduced in size, and that this size reduction coincides with the loss of helices from tRNA and LSU rRNA secondary structures. We infer that some of the helices that have been lost in the LSU rRNA are in regions that would normally contact the missing tRNA helices during protein-synthesis, suggesting coevolution of tRNA and rRNA secondary structures. We find wide variation in nucleotide skew in all mitochondrial protein-coding genes among taxa within chelicerates and conclude that the skew cannot be predicted at the deepest phylogenetic levels. We also find that there have been rearrangements of genes within the mt genomes of pseudoscorpions. However, these rearrangements appear not to have affected the distribution of nucleotide skew in the genome, as would be predicted if rearrangements influenced the orientation of the origin of replication.

Our phylogenetic analyses unfailingly place pseudoscorpions as the sister lineage to Acariformes, and not to lineages with which they apparently share more anatomical features. Similarities due to shared nucleotide or amino acid skew are likely not responsible for incorrectly placing Pseudoscorpiones with Acariformes, as these lineages do not share the same patterns of nucleotide skew. Indeed, nucleotide skew appears to have little effect on phylogeny reconstruction using amino acids, as lineages with reversed skew are scattered across our phylogenetic trees. We find several morphological features at the molecular level that Pseudoscorpiones do share with Acariformes. These include (1) the propensity for the evolutionary loss of sequence to encode D- or T-arms from tRNA genes, (2) a propensity for genome rearrangements, and (3) reduction in size of rRNA genes. Further sequencing of pseudoscorpion and acariform mitochondrial genomes and analyses of their tRNA and rRNA secondary structures will allow a more complete understanding of the degree to which the shared genomic changes they have undergone may be due to shared ancestry or to convergent evolution.

## Methods

### Taxon selection, nucleic acid extraction, and sequencing

We used two taxa for this study, *Pseudogarypus banksi *from the pseudoscorpion superfamily Feaelloidea and *Paratemnoides elongatus *from the superfamily Cheliferoidea. Phylogenetic analyses of molecular data place the superfamily Feaelloidea as sister to the other pseudoscorpions [59]; therefore these taxa are from substantially divergent lineages. The specimens were a gift from Jeff Shultz, and first identified by him, and then subsequently by Mark Harvey, an expert on pseudoscorpions.

Total genomic DNA was extracted from the legs of one adult for each specimen using the Qiagen™ DNAeasy kit. A region of the mtDNA cytochrome oxidase 1 gene was amplified with the primers HCO2198 and LCO1490 [60], and a region of cytochrome b was amplified with the primers CytbF and CytbR [61]. The PCR products were cleaned and sequenced, and primers were designed that faced outward from these regions. The *Paratemnoides*-specific primers we designed were Paret3CO1-UF **(**5'-CTC TGT TTG TAT GGT CCG TG-3') paired with ParetCytb2-LR **(**5'-GTT TGA TAC TGC AAA GTT TCC TC-3'), and ParetND4L-LR (5'-ACA TAG AAA TTA ATA AAC CAA CCA C-3') paired with ParetCO2-LR **(**5'-GTA AAA CTA TAT TAT TAA ATG TGT G-3'). The *Pseudogarypus-*specific primers we designed were PseCO1-UF (5'-CTG TAT TAG CGG GAG CAA TCA CCA T-3') paired with PseCob-LR (5'-GGG GGT GAG TAT AGG GTT GGC TTC-3') and PseCO1-LR (5'-GTC CAC CCT GTT CCA CAT CCT ATC TC-3') paired with Pse2Cob-UF (5'- ACT CAC CCC CAC CCA TAT TAA ACC -3').

Long PCR amplification of the two halves of the genomes was performed using the Takara™ LA Taq DNA polymerase kit. A 100-μl reaction contained final concentrations of 0.16 mM of each dNTP, 0.4 mM of each primer, 1× Takara ™ polymerase buffer, 1 μl of mitochondrial DNA (concentration not determined) and 2.5 Units of Takara ™ polymerase. The reactions were cycled at 92°C for 30 sec, 50-58°C (depending on the primers) for 25 sec, and 68°C for 12 min, for 37 cycles, followed by a final extension at 72°C for 15 min. The PCR products were electrophoresed in a 0.8% agarose gel to estimate size and concentration, cleaned, and resuspended in water.

The long PCR products were processed and sequenced at the DOE Joint Genome Institute, using methods previously described [5]. Sequences were processed using Phred, trimmed for quality, and assembled using Phrap. Quality scores were assigned automatically, and the electropherograms and assembly were viewed and verified for accuracy using Sequencher™ (GeneCodes).

### Sequence annotation and inferences of secondary structures

Protein-encoding genes were identified by similarity of inferred amino acid sequences to those of other arthropod mtDNAs. Once the protein-coding gene boundaries had been determined, the remaining regions were searched for tRNAs with the use of the program tRNAscan-SE 1.21 [62]. Any regions still not identified as coding for a gene were searched for conserved tRNA anticodon motifs. Potential tRNA genes were compared to the tRNA gene sequences from other chelicerates, using the methods outlined by Masta and Boore [5].

Ribosomal RNA gene locations were inferred based on sequence similarity to other chelicerates, and by inferring regions of conserved secondary structures of the SSU rRNA and LSU rRNA. The entire secondary structures of the LSU rRNAs for both pseudoscorpions were inferred by comparisons with conserved regions in Archaea, Bacteria, and Eucarya [40] and by using the mt LSU rRNA structure from the harvestman *Phalangium opilio *[14]. The rRNA helices were numbered following the scheme of Wuyts et al. [40].

### Sequence alignments

We added the new pseudoscorpion sequences to our existing alignments of mt genome sequences of chelicerates. Additionally, sequences from other arthropod mt genomes were downloaded from GenBank. These included sequences from Myriapoda and Pancrustacea, which were used to root the phylogenetic trees. A full list of taxa used in this study, along with their mt genome GenBank numbers, is provided in Additional file 1: Table S1.

Using the annotated gene boundary information, protein-coding genes were individually excised from the genomic sequence and put into an individual file for each gene. The program SeaView [63] was used to view the sequences as amino acids as they would be translated with the invertebrate mitochondrial genetic code. For three of the taxa available on GenBank, some genes likely contained sequencing errors, as stop codons were present within the genes. We corrected this by adding extra "N" characters to help place nucleotide sequences into the correct reading frame. For these taxa and genes (*Stylochyrus rarior CO3 *and *ND4, Bothropolys *sp. *Cytb, Daphnia pulex CO1*), the correct reading frame was identified when no internal stop codons were found and the amino acid sequences appeared to be relatively similar to the others in the alignment. CLUSTAL W version 2.0.12 [64] was used to align each of these 13 genes, using the Gonnet series protein matrix, with a gap opening of 10 and gap extension penalty of 0.2. The nucleotide sequence was then aligned using the amino acid alignment information, using a scripted pipeline.

In an effort to assure that only homologous regions of the sequence alignments were used in phylogenetic analyses, the program Gblocks [65] was used to remove regions that were ambiguously aligned or had poorly conserved amino acids. This method has been shown to improve phylogenetic signal, when used in conjunction with maximum likelihood methods [66]. The aligned amino acids were trimmed with Gblocks version 0.91b, using default parameters, with the exceptions of "type of sequence", which was set to "codons", and "allowed gap positions", which was set to "with half." This latter setting allowed sites that are without gaps in at least half the taxa to be retained. After Gblocks trimming of each of the 13 protein-coding sequence alignments, the 13 datasets were concatenated into a single alignment file that was used in subsequent phylogenetic analyses.

### Phylogenetic analyses

Phylogenetic analyses using maximum likelihood were performed on the Gblocks-reduced amino acid sequence alignments implemented in the program RAxML 7.2.8 [67]. We employed several different models of evolution in different analyses. In one set of analyses, the general time-reversible (GTR) model was used, with the gamma-distributed model for rate heterogeneity. Other analyses used the mtART [68] or the mtREV models of evolution with the gamma-distributed model for rate heterogeneity. These models were selected because they had previously been found to perform well with arthropod mitogenomic data [43,68]. For each of these different models of evolution, we performed an additional likelihood analysis that also estimated the proportion of invariable sites (I). For 5 of these different models of evolution, 10 replicate runs were performed. Due to time constraints, only 2 runs were performed for the GTR + G + I model of evolution. After each analysis, a majority-rule consensus of the 10 best trees was constructed using SumTrees 3.0 [69].

For each model of evolution employed, 1000 bootstrap replicates were performed in 10 separate runs, with 100 bootstrap replicates in each run. Each bootstrap analysis was conducted with random seed values.

Bayesian analyses were performed using PhyloBayes 3.2f [70]. A site-homogeneous model was used for site-specific frequencies in one set of analyses. The same three models of evolution (GTR, mtREV, and mtART) as used in likelihood analyses were used for Bayesian inference. However, in PhyloBayes analyses, site-specific rates across sites were modeled using a Dirichlet process. Each run consisted of two separate chains of at least 3 million generations, with tree sampling taken every 100 generations. Burn-in was calculated after one-fourth of the trees were produced, with the remaining trees used to produce a consensus tree and calculate posterior probabilities. We also performed analyses using the CAT site-heterogeneous mixture model, as suggested by Lartillot et al. [71]. We ran 34 chains in PhyloBayes, applying the CAT model for both frequency and rates site-heterogeneity. Each chain was run for 300,000 to 500,000 generations, until the chains stabilized.

During exploratory analyses to determine whether certain taxa on long branches influenced our phylogenetic results, we tried removing or adding specific taxa to our analyses. Each time a taxon was added or removed from the dataset, the entire alignment and Gblock trimming process as described above was repeated, followed by phylogenetic analyses.

To overcome the potential problems that nucleotide skew can have in creating misleading phylogenetic inferences, multiple analyses were undertaken to counter the effect at both the nucleotide and amino acid levels. Four recoded datasets were generated. We recoded mitochondrial nucleotides as either purines or pyrimidines (RY recoding), following the suggestions of Phillips and Penny [44]. For one dataset we RY recoded at 3^rd ^codon positions only, and for another dataset, we RY recoded 1^st ^and 3^rd ^codon positions. We also implemented a variation on RY recoding as proposed by Hassanin et al. [46], termed the Neutral Transitions Excluded (NTE) method, whereby selected codons are RY recoded. For all RY recoding, maximum likelihood analyses were conducted in RAxML using a GTR plus gamma model of evolution.

In a 4^th ^recoded data set, we aimed to overcome possible saturation and effects of amino acid skew by recoding amino acids into physiochemical groups. We categorized amino acids into the following six functional groups: hydrophobic (valine, leucine, isoleucine, and methionine); aromatic (phenylalanine, tyrosine, and tryptophan); small/neutral (serine, threonine, alanine, proline, and glycine); acidic/amides (aspartate, glutamate, asparagine, and glutamine); basic (histidine, arginine, and lysine); and sulfhydryl (cysteine). Both Bayesian and maximum likelihood analyses were performed on this recoded dataset.

### Hypothesis testing

To evaluate whether alternative phylogenetic hypotheses were compatible with our data, we used Approximately Unbiased (AU) tests [72] to assess alternative tree topologies. To evaluate the possibility of arachnid monophyly, we constrained all arachnids to be monophyletic, but excluded pycnogonids and xiphosurans from this clade. We did not enforce any further constraints on that tree topology. We also evaluated the hypotheses of a monophyletic Acari, and of a sister-group relationship between Solifugae and Pseudoscorpiones. Constraint trees were generated in Newick format, and likelihood analyses on the constraint trees were run in RAxML, using the MtART + gamma model of evolution. RAxML was used to generate per-site log likelihood scores, which were passed to the program CONSEL [73] to determine the statistical support for the alternative topologies.

### Nucleotide skew analyses

Nucleotide frequencies were determined for the entire mitochondrial genomes, for each of the 13 protein-coding genes, and for 1^st^, 2^nd^, and 3rd codon positions. Nucleotide skew was calculated following Perna and Kocher [74] where AT skew = (A - T)/(A + T) and CG skew = (C - G)/(C + G). Most genes are located on one strand, termed the major strand, and skew was calculated directly for these genes. For genes encoded by the opposite strand, the complement identity of the nucleotide was used in skew calculations. The total skew across all protein-coding genes for each of the codon positions was calculated by summing across all genes. The nucleotide skew at 3rd positions of codons was graphed for the arthropods used in this study. For the depiction in Figure 6 of taxa that did not possess skew (i.e. with lines colored purple), we considered values within the range of -0.2 to +0.2 to be non-skewed and values outside of that range to be skewed (see Additional file 3).

## Abbreviations

A: adenine; T: thymine; C: cytosine; G: guanine; Mt: mitochondrial; T-arm: TΨC-arm or pseudouridine-arm of a tRNA secondary structure; D-arm: dihydrouridine-arm of a tRNA secondary structure; rRNA: ribosomal RNA; *srRNA*: small rRNA; subunit or 12S (gene); *LSU rRNA*: large subunit ribosomal RNA or 16S (gene); *Atp6 *and *8*: ATPase subunit 6 and 8; *CO1*-*3*: cytochrome oxidase subunits I-III; *Cytb*: cytochrome b; *ND1-6*: NADH dehydrogenase subunits 1-6; lnr: large non-coding region/control region; bp: basepairs; nt: nucleotides

## Authors' contributions

SEM participated in obtaining the sequence data, annotating the genomic data, determining secondary structures of RNAs, and drafting the manuscript. SO participated in phylogenetic analyses, analyses of nucleotide skews, and determining secondary structures of rRNAs. Both authors read and approved the final manuscript.

## Supplementary Material

Additional file 1 **List of taxa used in this study**. List of taxa used in this study, arranged by their Linnaean taxonomy, with GenBank accession numbers for their mitochondrial genome sequence. Click here for file

Additional file 2 **Nucleotide use and AT and CG skew in chelicerate mitochondrial protein-coding genes**. Genes are arranged in the linearized order that they occur in the mitochondrial genome. Orange shading indicates genes found on the major strand, whereas gold indicates genes located on the minor strand. Nucleotide use and skew is shown for each of the three codon positions for each gene. The red to blue shading in the AT skew and CG skew columns indicate the degree of skew they possess, with the darker shading indicating greater skew. Blue indicates and excess of that nucleotide, red indicates a deficiency of that nucleotide. Click here for file

Additional file 3 **Graph of nucleotide skew at 3^rd ^codon positions of mitochondrial protein-coding genes of arthropods**. CG skew is plotted along the Y-axis, and AT skew is plotted on the X-axis. Skew was calculated following Perna and Kocher [65], and described in the Methods. The red square bounds the taxa we considered to not have a pronounced CG or AT nucleotide skew, that is, within the range of -0.2 to +0.2. These taxa have purple branches leading to them in Figure 6. The two purple dots depict the pseudoscorpions, the dark green dots depict the other chelicerates, and the light green dots depict the arthropod outgroup taxa analyzed in this study. The taxa used to create this graph are given in Additional file 1. Click here for file
