# Supplementary material for: Pseudoscorpion mitochondria show rearranged genes and genome-wide reductions of RNA gene sizes and inferred structures, yet typical nucleotide composition bias
Source: BMC Evol Biol. 2012 Mar 12;12:31. doi: 10.1186/1471-2148-12-31 (PMC3325882; doi:10.1186/1471-2148-12-31)
Supplement: Additional file 2 — Nucleotide use and AT and CG skew in chelicerate mitochondrial protein-coding genes. Genes are arranged in the linearized order that they occur in the mitochondrial genome. Orange shading indicates genes found on the major strand, whereas gold indicates genes located on the minor strand. Nucleotide use and skew is shown for each of the three codon positions for each gene. The red to blue shading in the AT skew and CG skew columns indicate the degree of skew they possess, with the darker shading indicating greater skew. Blue indicates and excess of that nucleotide, red indicates a deficiency of that nucleotide. [file 1471-2148-12-31-S2.PDF]

ACARIFORMES *Unionicola foili*

		A	T	C	G	ATskew	CGskew
ND2	1	144	98	46	25	0.1901	0.2958
major	2	62	154	60	37	-0.4259	0.2371
	3	130	100	68	15	0.1304	0.6386
CO1	1	170	139	84	119	0.1003	-0.1724
major	2	91	212	126	83	-0.3993	0.2057
	3	215	176	105	16	0.0997	0.7355
CO2	1	80	61	42	38	0.1348	0.05
major	2	55	85	52	29	-0.2143	0.284
	3	106	63	41	11	0.2544	0.5769
ATP8	1	19	19	6	5	0	0.0909
major	2	14	19	12	4	-0.1515	0.5
	3	28	10	9	2	0.4737	0.6364
ATP6	1	97	62	42	20	0.2201	0.3548
major	2	44	115	48	14	-0.4465	0.5484
	3	86	79	47	9	0.0424	0.6786
CO3	1	92	78	43	47	0.0824	-0.0444
major	2	52	100	64	44	-0.3158	0.1852
	3	124	79	53	4	0.2217	0.8596
ND5	1	96	292	35	121	-0.5052	-0.5513
minor	2	69	301	93	81	-0.627	0.069
	3	91	348	25	80	-0.5854	-0.5238
ND4L	1	22	42	7	17	-0.3125	-0.4167
minor	2	12	54	8	14	-0.6364	-0.2727
	3	16	54	4	14	-0.5429	-0.5556
ND3	1	53	31	9	16	0.2619	-0.28
major	2	31	50	14	14	-0.2346	0
	3	54	25	26	4	0.3671	0.7333
ND4	1	104	214	43	72	-0.3459	-0.2522
minor	2	71	250	64	48	-0.5576	0.1429
	3	105	231	39	58	-0.375	-0.1959
ND6	1	80	47	12	8	0.2598	0.2
major	2	31	80	27	9	-0.4414	0.5
	3	80	42	23	2	0.3115	0.84
CytB	1	131	117	62	55	0.0565	0.0598
major	2	78	152	88	47	-0.3217	0.3037
	3	170	119	58	18	0.1765	0.5263
ND1	1	52	160	24	60	-0.5094	-0.4286
minor	2	44	158	46	48	-0.5644	-0.0213
	3	51	200	8	37	-0.5936	-0.6444
all	1	1574	926	616	442	0.2592	0.1645
	2	1221	1163	682	492	0.0243	0.1618
	3	1826	956	619	157	0.3127	0.5954

ACARIFORMES *Leptotrombidium pallidum*

		A	T	C	G	ATskew	CGskew
ND2	1	99	114	42	40	-0.0704	0.0244
major	2	51	163	46	35	-0.5234	0.1358
	3	111	135	30	19	-0.0976	0.2245
CO1	1	140	146	89	136	-0.021	-0.2089
major	2	84	210	132	85	-0.4286	0.2166
	3	198	227	58	28	-0.0682	0.3488
CO2	1	65	68	41	42	-0.0226	-0.012
major	2	51	83	51	31	-0.2388	0.2439
	3	88	94	26	8	-0.033	0.5294
ND4L	1	23	37	9	16	-0.2333	-0.28
minor	2	11	54	11	9	-0.6615	0.1
	3	29	37	9	10	-0.1212	-0.0526
ND1	1	88	104	56	45	-0.0833	0.1089
major	2	51	143	57	42	-0.4742	0.1515
	3	115	128	27	23	-0.0535	0.08
ND4	1	119	158	57	74	-0.1408	-0.1298
minor	2	62	204	71	71	-0.5338	0
	3	167	174	38	29	-0.0205	0.1343
ND6	1	61	46	16	20	0.1402	-0.1111
major	2	26	84	19	14	-0.5273	0.1515
	3	65	60	11	7	0.04	0.2222
CytB	1	109	110	58	74	-0.0046	-0.1212
major	2	65	152	85	49	-0.4009	0.2687
	3	127	169	30	25	-0.1419	0.0909
ATP8	1	23	19	5	3	0.0952	0.25
major	2	16	19	9	6	-0.0857	0.2
	3	19	23	7	1	-0.0952	0.75
ATP6	1	70	77	25	36	-0.0476	-0.1803
major	2	42	99	50	17	-0.4043	0.4925
	3	88	84	20	16	0.0233	0.1111
CO3	1	63	94	41	61	-0.1975	-0.1961
major	2	52	109	59	39	-0.354	0.2041
	3	102	106	36	15	-0.0192	0.4118
ND5	1	170	184	66	92	-0.0395	-0.1646
minor	2	88	252	99	73	-0.4824	0.1512
	3	204	212	55	41	-0.0192	0.1458
ND3	1	29	43	13	17	-0.1944	-0.1333
major	2	23	49	15	15	-0.3611	0
	3	37	47	10	8	-0.119	0.1111
all	1	1126	1133	568	606	-0.0031	-0.0324
	2	971	1272	676	514	-0.1342	0.1361
	3	1373	1473	335	252	-0.0351	0.1414

ACARIFORMES *Walchia hayashii*

		A	T	C	G	ATskew	CGskew
ND2	1	180	67	37	27	0.4575	0.1563
major	2	68	152	60	31	-0.3818	0.3187
	3	199	66	42	4	0.5019	0.8261
CO1	1	187	133	81	112	0.1688	-0.1606
major	2	95	210	131	77	-0.377	0.2596
	3	304	117	85	7	0.4442	0.8478
ND1	1	65	137	27	67	-0.3564	-0.4255
minor	2	39	166	42	49	-0.6195	-0.0769
	3	47	184	5	60	-0.5931	-0.8462
CO2	1	95	55	41	29	0.2667	0.1714
major	2	54	77	63	26	-0.1756	0.4157
	3	141	36	40	3	0.5932	0.8605
ATP8	1	33	8	6	1	0.6098	0.7143
major	2	16	19	10	3	-0.0857	0.5385
	3	36	7	4	1	0.6744	0.6
ATP6	1	108	44	32	30	0.4211	0.0323
major	2	46	91	60	17	-0.3285	0.5584
	3	134	35	43	2	0.5858	0.9111
CO3	1	99	73	37	51	0.1512	-0.1591
major	2	56	110	69	25	-0.3253	0.4681
	3	162	49	47	2	0.5355	0.9184
ND4L	1	24	37	5	24	-0.2131	-0.6552
minor	2	10	52	9	19	-0.6774	-0.3571
	3	30	46	3	11	-0.2105	-0.5714
ND4	1	92	202	37	87	-0.3741	-0.4032
minor	2	58	233	63	64	-0.6014	-0.0079
	3	128	229	17	44	-0.2829	-0.4426
ND6	1	65	50	16	14	0.1304	0.0667
major	2	25	78	35	7	-0.5146	0.6667
	3	86	37	18	4	0.3984	0.6364
CytB	1	136	102	56	67	0.1429	-0.0894
major	2	74	153	93	41	-0.348	0.3881
	3	196	102	54	9	0.3154	0.7143
ND5	1	122	260	39	114	-0.3613	-0.4902
minor	2	79	294	90	72	-0.5764	0.1111
	3	113	348	10	64	-0.5098	-0.7297
ND3	1	55	24	13	18	0.3924	-0.1613
major	2	25	50	28	7	-0.3333	0.6
	3	74	20	14	2	0.5745	0.75
all	1	1594	859	611	457	0.2996	0.1442
	2	1204	1126	753	438	0.0335	0.2645
	3	2139	787	526	69	0.4621	0.7681

ACARIFORMES *Ascoschoengastia* sp.

		A	T	C	G	ATskew	CGskew
ND2	1	143	83	39	44	0.2655	-0.0602
major	2	59	171	48	31	-0.487	0.2152
	3	145	77	49	38	0.3063	0.1264
CO1	1	176	156	77	106	0.0602	-0.1585
major	2	91	212	130	82	-0.3993	0.2264
	3	221	151	99	44	0.1882	0.3846
CytB	1	113	122	59	68	-0.0383	-0.0709
minor	2	73	168	71	50	-0.3942	0.1736
	3	126	107	73	56	0.0815	0.1318
ND6	1	66	45	15	19	0.1892	-0.1176
minor	2	23	83	24	15	-0.566	0.2308
	3	47	59	18	21	-0.1132	-0.0769
ND4	1	147	154	50	67	-0.0233	-0.1453
major	2	68	214	78	58	-0.5177	0.1471
	3	155	131	73	59	0.0839	0.1061
ND4L	1	33	27	9	17	0.1	-0.3077
major	2	15	49	11	11	-0.5313	0
	3	30	30	14	12	0	0.0769
ND1	1	90	110	34	60	-0.1	-0.2766
minor	2	42	160	53	39	-0.5842	0.1522
	3	104	98	40	52	0.0297	-0.1304
ND5	1	188	198	56	92	-0.0259	-0.2432
major	2	100	279	92	63	-0.4723	0.1871
	3	211	172	97	54	0.1018	0.2848
CO3	1	79	93	35	53	-0.0814	-0.2045
minor	2	53	118	58	31	-0.3801	0.3034
	3	78	107	31	44	-0.1568	-0.1733
ATP6	1	76	79	25	36	-0.0194	-0.1803
minor	2	39	109	44	24	-0.473	0.2941
	3	70	82	32	32	-0.0789	0
ATP8	1	26	13	5	4	0.3333	0.1111
minor	2	15	22	7	4	-0.1892	0.2727
	3	22	14	4	8	0.2222	-0.3333
CO2	1	68	79	32	41	-0.0748	-0.1233
minor	2	48	94	50	28	-0.3239	0.2821
	3	68	92	25	35	-0.15	-0.1667
ND3	1	42	39	11	18	0.037	-0.2414
major	2	21	59	22	8	-0.475	0.4667
	3	44	35	21	10	0.1139	0.3548
all	1	1270	1175	523	549	0.0389	-0.0243
	2	1108	1277	572	560	-0.0709	0.0106
	3	1365	1111	601	440	0.1026	0.1547

ACARIFORMES *Steganacarus magnus*

		A	T	C	G	ATskew	CGskew
CO1	1	146	177	70	118	-0.096	-0.2553
major	2	96	218	115	82	-0.3885	0.1675
	3	144	299	18	50	-0.3499	-0.4706
CO2	1	70	87	25	40	-0.1083	-0.2308
major	2	50	94	49	29	-0.3056	0.2564
	3	66	115	15	26	-0.2707	-0.2683
ATP8	1	21	19	4	6	0.05	-0.2
major	2	13	24	8	5	-0.2973	0.2308
	3	19	28	0	3	-0.1915	-1
ATP6	1	69	88	31	28	-0.121	0.0508
major	2	37	126	40	13	-0.546	0.5094
	3	63	134	11	8	-0.3604	0.1579
CO3	1	72	107	29	54	-0.1955	-0.3012
major	2	51	120	55	36	-0.4035	0.2088
	3	79	143	15	25	-0.2883	-0.25
ND3	1	35	44	10	23	-0.1139	-0.3939
major	2	28	59	11	14	-0.3563	-0.12
	3	31	63	6	12	-0.3404	-0.3333
ND5	1	192	204	45	87	-0.0303	-0.3182
minor	2	91	261	100	77	-0.483	0.1299
	3	224	227	47	30	-0.0067	0.2208
ND4	1	133	168	51	68	-0.1163	-0.1429
minor	2	73	223	71	53	-0.5068	0.1452
	3	150	199	40	31	-0.1404	0.1268
ND4L	1	27	41	7	14	-0.2059	-0.3333
minor	2	14	47	18	10	-0.541	0.2857
	3	30	46	9	4	-0.2105	0.3846
ND6	1	45	59	12	19	-0.1346	-0.2258
major	2	27	66	30	12	-0.4194	0.4286
	3	61	54	11	9	0.0609	0.1
CytB	1	121	113	50	70	0.0342	-0.1667
major	2	70	163	73	48	-0.3991	0.2066
	3	124	166	46	18	-0.1448	0.4375
ND2	1	149	104	31	35	0.1779	-0.0606
major	2	64	165	52	38	-0.441	0.1556
	3	143	115	37	24	0.1085	0.2131
ND1	1	66	126	36	70	-0.3125	-0.3208
minor	2	55	159	40	43	-0.486	-0.0361
	3	82	166	24	26	-0.3387	-0.04
all	1	1267	1216	501	532	0.0205	-0.03
	2	1126	1268	616	506	-0.0593	0.098
	3	1368	1603	250	295	-0.0791	-0.0826

ACARIFORMES *Dermatophagoides farinae*

		A	T	C	G	ATskew	CGskew
CO1	1	137	168	64	151	-0.1016	-0.4047
major	2	95	219	117	89	-0.3949	0.1359
	3	91	308	40	81	-0.5439	-0.3388
CO2	1	58	103	32	60	-0.2795	-0.3043
major	2	66	104	52	31	-0.2235	0.253
	3	47	150	14	42	-0.5228	-0.5
ATP8	1	17	21	9	5	-0.1053	0.2857
major	2	7	29	10	6	-0.6111	0.25
	3	13	30	2	7	-0.3953	-0.5556
ATP6	1	57	90	32	48	-0.2245	-0.2
major	2	38	119	39	31	-0.5159	0.1143
	3	42	137	8	40	-0.5307	-0.6667
CO3	1	44	111	32	75	-0.4323	-0.4019
major	2	51	113	42	56	-0.378	-0.1429
	3	62	135	18	47	-0.3706	-0.4462
ND3	1	23	55	6	26	-0.4103	-0.625
major	2	11	64	16	19	-0.7067	-0.0857
	3	24	57	11	18	-0.4074	-0.2414
ND1	1	100	113	36	51	-0.061	-0.1724
minor	2	58	145	45	52	-0.4286	-0.0722
	3	125	97	46	32	0.1261	0.1795
ND6	1	67	53	14	13	0.1167	0.037
minor	2	30	67	35	15	-0.3814	0.4
	3	67	41	26	13	0.2407	0.3333
ND4L	1	24	45	6	16	-0.3043	-0.4545
major	2	12	53	10	16	-0.6308	-0.2308
	3	13	55	3	20	-0.6176	-0.7391
ND4	1	95	202	43	90	-0.3603	-0.3534
major	2	62	255	51	62	-0.6088	-0.0973
	3	84	249	26	71	-0.4955	-0.4639
ND5	1	148	234	47	115	-0.2251	-0.4198
major	2	89	287	91	77	-0.5266	0.0833
	3	118	321	26	79	-0.4624	-0.5048
ND2	1	144	94	39	33	0.2101	0.0833
minor	2	71	147	64	28	-0.3486	0.3913
	3	172	69	55	14	0.4274	0.5942
CytB	1	134	94	62	74	0.1754	-0.0882
minor	2	75	160	88	41	-0.3617	0.3643
	3	182	78	76	28	0.4	0.4615
all	1	957	1474	442	737	-0.2127	-0.2502
	2	950	1477	564	619	-0.2171	-0.0465
	3	779	1988	235	608	-0.4369	-0.4425

PSEUDOSCORPIONES *Pseudogarypus banksi*

		A	T	C	G	ATskew	CGskew
ND2	1	145	111	33	36	0.1328	-0.0435
major	2	66	159	66	34	-0.4133	0.32
	3	148	135	28	14	0.0459	0.3333
CO1	1	165	158	71	118	0.0217	-0.2487
major	2	95	219	125	73	-0.3949	0.2626
	3	205	209	69	28	-0.0097	0.4227
CO2	1	89	66	35	38	0.1484	-0.0411
major	2	66	93	47	22	-0.1698	0.3623
	3	96	97	32	3	-0.0052	0.8286
ATP8	1	23	23	5	0	0	1
major	2	18	25	4	4	-0.1628	0
	3	20	20	8	3	0	0.4545
ATP6	1	98	71	24	21	0.1598	0.0667
major	2	38	116	37	23	-0.5065	0.2333
	3	91	88	27	8	0.0168	0.5429
CO3	1	79	95	42	42	-0.092	0
major	2	55	121	51	31	-0.375	0.2439
	3	105	108	31	14	-0.0141	0.3778
ND3	1	45	52	11	9	-0.0722	0.1
major	2	26	63	18	10	-0.4157	0.2857
	3	52	54	10	1	-0.0189	0.8182
CytB	1	157	109	47	47	0.1805	0
major	2	74	162	77	47	-0.3729	0.2419
	3	150	155	46	9	-0.0164	0.6727
ND4L	1	29	43	3	17	-0.1944	-0.7
minor	2	20	53	7	12	-0.4521	-0.2632
	3	37	40	3	12	-0.039	-0.6
ND6	1	70	54	19	9	0.129	0.3571
major	2	32	86	29	5	-0.4576	0.7059
	3	57	68	23	4	-0.088	0.7037
ND4	1	132	192	37	78	-0.1852	-0.3565
minor	2	78	250	49	62	-0.5244	-0.1171
	3	178	206	13	42	-0.0729	-0.5273
ND5	1	174	239	32	110	-0.1574	-0.5493
minor	2	116	294	60	83	-0.4341	-0.1608
	3	200	257	25	73	-0.1247	-0.4898
ND1	1	76	128	26	66	-0.2549	-0.4348
minor	2	54	163	35	44	-0.5023	-0.1139
	3	101	139	5	51	-0.1583	-0.8214
all	1	1473	1150	558	418	0.1231	0.1434
	2	1230	1312	655	400	-0.0323	0.2417
	3	1566	1450	452	130	0.0385	0.5533

PSEUDOSCORPIONES *Paratemnoides elongatus*

		A	T	C	G	ATskew	CGskew
ND2	1	123	109	30	39	0.0603	-0.1304
major	2	54	155	53	40	-0.4833	0.1398
	3	141	110	28	23	0.1235	0.098
CO1	1	159	164	72	115	-0.0155	-0.2299
major	2	90	232	111	77	-0.441	0.1809
	3	187	233	56	32	-0.1095	0.2727
CO2	1	82	66	35	41	0.1081	-0.0789
major	2	61	89	38	36	-0.1867	0.027
	3	86	94	29	15	-0.0444	0.3182
ATP8	1	24	12	11	3	0.3333	0.5714
major	2	16	24	6	4	-0.2	0.2
	3	23	16	9	2	0.1795	0.6364
ATP6	1	91	64	36	26	0.1742	0.1613
major	2	40	106	44	27	-0.4521	0.2394
	3	85	98	23	11	-0.071	0.3529
CO3	1	83	90	46	44	-0.0405	0.0222
major	2	57	114	54	38	-0.3333	0.1739
	3	106	108	38	11	-0.0093	0.551
ND3	1	50	38	12	15	0.1364	-0.1111
major	2	25	64	16	10	-0.4382	0.2308
	3	42	50	17	6	-0.087	0.4783
ND5	1	176	196	37	129	-0.0538	-0.5542
minor	2	113	266	76	83	-0.4037	-0.044
	3	202	231	28	77	-0.067	-0.4667
ND4	1	143	148	42	89	-0.0172	-0.3588
minor	2	79	224	54	65	-0.4785	-0.0924
	3	173	170	21	58	0.0087	-0.4684
ND4L	1	28	30	6	22	-0.0345	-0.5714
minor	2	22	50	4	10	-0.3889	-0.4286
	3	30	39	4	13	-0.1304	-0.5294
ND6	1	64	47	24	13	0.1532	0.2973
major	2	21	86	30	11	-0.6075	0.4634
	3	61	56	23	8	0.0427	0.4839
CytB	1	124	113	65	56	0.0464	0.0744
major	2	71	163	79	45	-0.3932	0.2742
	3	146	139	62	11	0.0246	0.6986
ND1	1	91	108	26	69	-0.0854	-0.4526
minor	2	51	163	30	50	-0.5234	-0.25
	3	106	126	8	54	-0.0862	-0.7419
all	1	1282	1141	640	463	0.0582	0.1605
	2	1138	1298	639	452	-0.0657	0.1714
	3	1443	1415	487	180	0.0098	0.4603

PYCNOGONIDA *Achelia bituberculata*

		A	T	C	G	ATskew	CGskew
ND2	1	136	119	29	43	0.0667	-0.1944
major	2	63	184	42	38	-0.4899	0.05
	3	115	160	20	32	-0.1636	-0.2308
CO1	1	161	152	70	129	0.0288	-0.2965
major	2	93	222	112	85	-0.4095	0.1371
	3	185	255	30	42	-0.1591	-0.1667
CO2	1	83	57	35	48	0.1857	-0.1566
major	2	63	98	36	26	-0.2174	0.1613
	3	79	116	13	15	-0.1897	-0.0714
ATP8	1	23	19	8	2	0.0952	0.6
major	2	14	29	5	4	-0.3488	0.1111
	3	24	23	1	4	0.0213	-0.6
ATP6	1	99	72	20	31	0.1579	-0.2157
major	2	45	121	35	21	-0.4578	0.25
	3	83	112	13	14	-0.1487	-0.037
CO3	1	82	91	35	55	-0.052	-0.2222
major	2	55	112	47	49	-0.3413	-0.0208
	3	103	133	9	18	-0.1271	-0.3333
ND3	1	38	40	11	25	-0.0256	-0.3889
major	2	22	66	17	9	-0.5	0.3077
	3	40	53	5	16	-0.1398	-0.5238
ND5	1	215	206	57	90	0.0214	-0.2245
minor	2	116	288	92	72	-0.4257	0.122
	3	260	223	46	39	0.0766	0.0824
ND4	1	164	181	50	61	-0.0493	-0.0991
minor	2	101	227	66	62	-0.3841	0.0313
	3	192	211	22	31	-0.0471	-0.1698
ND4L	1	40	33	10	15	0.0959	-0.2
minor	2	25	51	6	16	-0.3421	-0.4545
	3	34	43	8	13	-0.1169	-0.2381
ND6	1	78	58	13	15	0.1471	-0.0714
major	2	37	91	18	18	-0.4219	0
	3	68	68	19	9	0	0.3571
CytB	1	134	112	51	64	0.0894	-0.113
major	2	72	174	74	41	-0.4146	0.287
	3	164	152	31	14	0.038	0.3778
ND1	1	87	134	26	64	-0.2127	-0.4222
minor	2	65	157	42	47	-0.4144	-0.0562
	3	118	146	19	28	-0.1061	-0.1915
all	1	1388	1226	502	555	0.062	-0.0501
	2	1187	1404	583	497	-0.0838	0.0796
	3	1484	1676	252	259	-0.0608	-0.0137

PYCNOGONIDA *Ammothea hilgendorfi*

		A	T	C	G	ATskew	CGskew
ND2	1	171	115	24	20	0.1958	0.0909
major	2	69	191	40	30	-0.4692	0.1429
	3	163	146	14	7	0.055	0.3333
CO1	1	176	154	63	120	0.0667	-0.3115
major	2	95	223	116	79	-0.4025	0.1897
	3	227	238	31	17	-0.0237	0.2917
CO2	1	99	58	34	32	0.2611	0.0303
major	2	64	97	35	27	-0.205	0.129
	3	97	109	13	4	-0.0583	0.5294
ATP8	1	30	18	5	0	0.25	1
major	2	15	25	8	5	-0.25	0.2308
	3	27	23	0	3	0.08	-1
ATP6	1	101	71	26	25	0.1744	0.0196
major	2	47	117	40	19	-0.4268	0.3559
	3	106	105	9	3	0.0047	0.5
CO3	1	86	98	33	45	-0.0652	-0.1538
major	2	56	116	46	43	-0.3488	0.0337
	3	121	126	8	7	-0.0202	0.0667
ND3	1	46	45	10	16	0.011	-0.2308
major	2	26	66	19	6	-0.4348	0.52
	3	53	56	5	3	-0.0275	0.25
ND5	1	232	226	33	81	0.0131	-0.4211
minor	2	124	299	81	68	-0.4137	0.0872
	3	236	280	17	39	-0.0853	-0.3929
ND4	1	157	205	30	61	-0.1326	-0.3407
minor	2	92	248	53	60	-0.4588	-0.0619
	3	184	230	9	30	-0.1111	-0.5385
ND4L	1	36	42	4	16	-0.0769	-0.6
minor	2	26	55	4	13	-0.358	-0.5294
	3	43	48	1	6	-0.0549	-0.7143
ND6	1	96	52	13	9	0.2973	0.1818
major	2	40	102	19	9	-0.4366	0.3571
	3	80	76	10	4	0.0256	0.4286
CytB	1	138	114	51	61	0.0952	-0.0893
major	2	76	172	72	44	-0.3871	0.2414
	3	157	163	34	10	-0.0188	0.5455
ND1	1	91	128	20	73	-0.1689	-0.5699
minor	2	64	160	35	53	-0.4286	-0.2045
	3	118	154	11	29	-0.1324	-0.45
all	1	1544	1241	490	415	0.1088	0.0829
	2	1250	1415	589	435	-0.0619	0.1504
	3	1743	1623	228	96	0.0357	0.4074

PYCNOGONIDA *Tanystylum orbiculare*

		A	T	C	G	ATskew	CGskew
ND2	1	151	125	21	32	0.0942	-0.2075
major	2	66	190	43	30	-0.4844	0.1781
	3	153	152	5	19	0.0033	-0.5833
CO1	1	166	159	64	123	0.0215	-0.3155
major	2	94	227	113	78	-0.4143	0.1832
	3	208	247	14	43	-0.0857	-0.5088
CO2	1	88	59	32	45	0.1973	-0.1688
major	2	63	94	39	28	-0.1975	0.1642
	3	83	126	0	15	-0.2057	-1
ATP8	1	21	20	6	6	0.0244	0
major	2	14	26	9	4	-0.3	0.3846
	3	28	23	1	1	0.098	0
ATP6	1	99	78	19	26	0.1186	-0.1556
major	2	45	117	38	22	-0.4444	0.2667
	3	92	109	6	15	-0.0846	-0.4286
CO3	1	80	101	31	50	-0.116	-0.2346
major	2	55	114	49	44	-0.3491	0.0538
	3	115	124	6	17	-0.0377	-0.4783
ND3	1	39	51	6	21	-0.1333	-0.5556
major	2	22	70	18	7	-0.5217	0.44
	3	52	60	1	4	-0.0714	-0.6
ND5	1	249	197	53	76	0.1166	-0.1783
minor	2	125	291	94	65	-0.399	0.1824
	3	260	266	32	17	-0.0114	0.3061
ND4	1	180	192	36	48	-0.0323	-0.1429
minor	2	91	248	62	55	-0.4631	0.0598
	3	205	203	35	13	0.0049	0.4583
ND4L	1	42	38	4	13	0.05	-0.5294
minor	2	20	54	10	13	-0.4595	-0.1304
	3	39	48	5	5	-0.1034	0
ND6	1	81	59	7	17	0.1571	-0.4167
major	2	33	100	21	10	-0.5038	0.3548
	3	70	81	4	9	-0.0728	-0.3846
CytB	1	138	113	46	67	0.0996	-0.1858
major	2	74	176	62	52	-0.408	0.0877
	3	151	183	8	22	-0.0958	-0.4667
ND1	1	112	112	28	56	0	-0.3333
minor	2	65	155	38	50	-0.4091	-0.1364
	3	135	137	23	13	-0.0074	0.2778
all	1	1402	1348	425	508	0.0196	-0.089
	2	1214	1415	575	479	-0.0765	0.0911
	3	1606	1744	93	240	-0.0412	-0.4414

PYCNOGONIDA *Nymphon gracile*

		A	T	C	G	ATskew	CGskew
CO2	1	74	67	34	53	0.0496	-0.2184
minor	2	60	95	44	29	-0.2258	0.2055
	3	77	129	6	16	-0.2524	-0.4545
CO1	1	149	164	69	131	-0.0479	-0.31
minor	2	96	221	111	85	-0.3943	0.1327
	3	159	301	9	44	-0.3087	-0.6604
ND2	1	141	124	12	49	0.0642	-0.6066
minor	2	50	184	54	38	-0.5726	0.1739
	3	119	178	3	26	-0.1987	-0.7931
ATP8	1	37	13	3	0	0.48	1
major	2	23	22	7	1	0.0222	0.75
	3	22	24	7	0	-0.0435	1
ATP6	1	90	60	32	23	0.2	0.1636
major	2	34	107	44	20	-0.5177	0.375
	3	111	79	13	2	0.1684	0.7333
CO3	1	94	80	37	52	0.0805	-0.1685
major	2	55	115	52	41	-0.3529	0.1183
	3	145	87	29	2	0.25	0.871
ND3	1	59	30	12	14	0.3258	-0.0769
major	2	26	60	23	6	-0.3953	0.5862
	3	49	50	13	3	-0.0101	0.625
ND5	1	153	264	37	104	-0.2662	-0.4752
minor	2	101	291	90	76	-0.4847	0.0843
	3	160	348	7	43	-0.3701	-0.72
ND4	1	139	205	36	81	-0.1919	-0.3846
minor	2	77	249	65	70	-0.5276	-0.037
	3	138	283	3	37	-0.3444	-0.85
ND4L	1	28	38	4	24	-0.1515	-0.7143
minor	2	17	51	11	15	-0.5	-0.1538
	3	33	49	0	12	-0.1951	-1
ND6	1	60	54	20	12	0.0526	0.25
major	2	31	75	32	8	-0.4151	0.6
	3	71	59	15	1	0.0923	0.875
CytB	1	134	116	50	63	0.072	-0.115
major	2	73	160	80	50	-0.3734	0.2308
	3	175	154	29	5	0.0638	0.7059
ND1	1	77	127	27	74	-0.2451	-0.4653
minor	2	61	148	44	52	-0.4163	-0.0833
	3	96	185	1	23	-0.3167	-0.9167
all	1	1463	1114	670	383	0.1354	0.2726
	2	1481	1001	603	545	0.1934	0.0505
	3	2046	1235	307	42	0.2472	0.7593

PYCNOGONIDA *Nymphon unguiculatum-charcoti* complex

		A	T	C	G	ATskew	CGskew
ND2	1	148	107	25	51	0.1608	-0.3421
major	2	61	178	60	31	-0.4895	0.3187
	3	148	145	5	33	0.0102	-0.7368
CO1	1	169	150	73	121	0.0596	-0.2474
major	2	98	215	121	79	-0.3738	0.21
	3	174	284	13	42	-0.2402	-0.5273
CO2	1	84	72	31	42	0.0769	-0.1507
major	2	65	90	49	25	-0.1613	0.3243
	3	78	129	6	16	-0.2464	-0.4545
ATP8	1	23	20	4	5	0.0698	-0.1111
major	2	14	25	8	5	-0.2821	0.2308
	3	22	25	0	5	-0.0638	-1
ATP6	1	81	67	33	24	0.0946	0.1579
major	2	30	103	46	26	-0.5489	0.2778
	3	88	95	5	17	-0.0383	-0.5455
CO3	1	96	79	36	50	0.0971	-0.1628
major	2	49	118	53	41	-0.4132	0.1277
	3	104	136	5	16	-0.1333	-0.5238
ND3	1	46	39	9	22	0.0824	-0.4194
major	2	25	65	17	9	-0.4444	0.3077
	3	44	62	2	8	-0.1698	-0.6
ND5	1	197	213	59	89	-0.039	-0.2027
minor	2	119	269	98	72	-0.3866	0.1529
	3	242	238	65	13	0.0083	0.6667
ND4	1	149	175	53	78	-0.0802	-0.1908
minor	2	85	225	84	61	-0.4516	0.1586
	3	216	177	50	12	0.0992	0.6129
ND4L	1	39	34	12	13	0.0685	-0.04
minor	2	20	53	12	13	-0.4521	-0.04
	3	43	41	12	2	0.0238	0.7143
ND6	1	60	61	18	21	-0.0083	-0.0769
major	2	32	84	27	17	-0.4483	0.2273
	3	56	87	3	14	-0.2168	-0.6471
CytB	1	127	118	53	67	0.0367	-0.1167
major	2	77	161	75	52	-0.3529	0.1811
	3	126	202	9	28	-0.2317	-0.5135
ND1	1	90	114	38	64	-0.1176	-0.2549
minor	2	61	150	49	46	-0.4218	0.0316
	3	124	137	34	11	-0.0498	0.5111
all	1	1370	1188	526	565	0.0711	-0.0357
	2	1148	1324	648	528	-0.0712	0.102
	3	1433	1790	86	340	-0.1108	-0.5962

PARASITIFORMES *Ixodes hexagonus*

		A	T	C	G	ATskew	CGskew
ND2	1	122	123	50	24	-0.0041	0.3514
major	2	52	166	79	22	-0.5229	0.5644
	3	121	112	77	9	0.0386	0.7907
CO1	1	161	147	88	117	0.0455	-0.1415
major	2	95	220	121	77	-0.3968	0.2222
	3	195	195	101	22	0	0.6423
CO2	1	87	65	37	36	0.1447	0.0137
major	2	53	94	55	23	-0.2789	0.4103
	3	85	81	54	5	0.0241	0.8305
ATP8	1	19	20	13	0	-0.0256	1
major	2	10	27	11	4	-0.4595	0.4667
	3	25	17	9	1	0.1905	0.8
ATP6	1	91	71	37	22	0.1235	0.2542
major	2	36	108	55	22	-0.5	0.4286
	3	94	73	47	7	0.1257	0.7407
CO3	1	84	89	46	39	-0.0289	0.0824
major	2	46	124	53	35	-0.4588	0.2045
	3	106	82	60	10	0.1277	0.7143
ND3	1	47	29	22	14	0.2368	0.2222
major	2	23	62	17	10	-0.4588	0.2593
	3	41	34	34	3	0.0933	0.8378
ND5	1	187	201	45	121	-0.0361	-0.4578
minor	2	108	288	75	83	-0.4545	-0.0506
	3	180	267	27	80	-0.1946	-0.4953
ND4	1	129	175	38	95	-0.1513	-0.4286
minor	2	75	239	47	76	-0.5223	-0.2358
	3	140	192	12	93	-0.1566	-0.7714
ND4L	1	37	34	4	17	0.0423	-0.619
minor	2	17	56	7	12	-0.5342	-0.2632
	3	31	33	2	26	-0.0313	-0.8571
ND6	1	70	43	22	7	0.2389	0.5172
major	2	23	92	23	4	-0.6	0.7037
	3	55	44	37	6	0.1111	0.7209
CytB	1	120	117	60	59	0.0127	0.0084
major	2	67	163	85	41	-0.4174	0.3492
	3	129	140	73	14	-0.0409	0.6782
ND1	1	106	117	18	72	-0.0493	-0.6
minor	2	63	159	33	58	-0.4324	-0.2747
	3	89	150	14	60	-0.2552	-0.6216
all	1	1328	1163	680	423	0.0662	0.233
	2	1147	1319	728	400	-0.0697	0.2908
	3	1493	1218	751	132	0.1014	0.701

PARASITIFORMES *Rhipicephalus sanguineus*

		A	T	C	G	ATskew	CGskew
ND2	1	153	122	20	19	0.1127	0.0256
major	2	67	174	49	24	-0.444	0.3425
	3	145	145	18	6	0	0.5
CO1	1	166	156	72	119	0.0311	-0.2461
major	2	96	223	119	75	-0.3981	0.2268
	3	221	235	44	13	-0.0307	0.5439
CO2	1	89	67	32	37	0.141	-0.0725
major	2	62	90	52	21	-0.1842	0.4247
	3	94	104	19	8	-0.0505	0.4074
ATP8	1	30	19	4	0	0.2245	1
major	2	17	27	7	2	-0.2273	0.5556
	3	23	27	2	1	-0.08	0.3333
ATP6	1	87	72	33	30	0.0943	0.0476
major	2	36	118	45	23	-0.5325	0.3235
	3	84	112	20	6	-0.1429	0.5385
CO3	1	72	101	37	49	-0.1676	-0.1395
major	2	54	113	53	39	-0.3533	0.1522
	3	112	121	17	9	-0.0386	0.3077
ND3	1	30	50	11	24	-0.25	-0.3714
major	2	24	66	16	9	-0.4667	0.28
	3	47	53	7	8	-0.06	-0.0667
ND1	1	124	109	33	48	0.0644	-0.1852
minor	2	69	160	44	41	-0.3974	0.0353
	3	145	136	26	7	0.032	0.5758
ND5	1	230	199	43	80	0.0723	-0.3008
minor	2	113	287	83	69	-0.435	0.0921
	3	249	246	39	18	0.0061	0.3684
ND4	1	160	163	47	64	-0.0093	-0.1532
minor	2	87	231	53	63	-0.4528	-0.0862
	3	194	194	24	22	0	0.0435
ND4L	1	36	34	8	14	0.0286	-0.2727
minor	2	14	58	5	15	-0.6111	-0.5
	3	41	45	2	4	-0.0465	-0.3333
ND6	1	73	57	9	11	0.1231	-0.1
major	2	30	88	22	10	-0.4915	0.375
	3	68	66	12	4	0.0149	0.5
CytB	1	128	122	43	61	0.024	-0.1731
major	2	72	166	74	42	-0.395	0.2759
	3	132	183	23	16	-0.1619	0.1795
all	1	1333	1316	467	481	0.0064	-0.0148
	2	1194	1348	625	430	-0.0606	0.1848
	3	1547	1675	213	162	-0.0397	0.136

PARASITIFORMES *Haemaphysalis flava*

		A	T	C	G	ATskew	CGskew
ND2	1	160	115	22	23	0.1636	-0.0222
major	2	68	184	46	22	-0.4603	0.3529
	3	139	143	30	8	-0.0142	0.5789
CO1	1	168	146	79	120	0.0701	-0.206
major	2	95	223	117	78	-0.4025	0.2
	3	222	215	54	22	0.016	0.4211
CO2	1	90	66	31	38	0.1538	-0.1014
major	2	60	92	49	24	-0.2105	0.3425
	3	99	90	26	10	0.0476	0.4444
ATP8	1	32	14	4	4	0.3913	0
major	2	13	31	8	2	-0.4091	0.6
	3	29	19	6	0	0.2083	1
ATP6	1	84	83	29	26	0.006	0.0545
major	2	38	117	44	23	-0.5097	0.3134
	3	72	114	19	17	-0.2258	0.0556
CO3	1	74	103	34	48	-0.1638	-0.1707
major	2	52	117	54	36	-0.3846	0.2
	3	95	127	13	24	-0.1441	-0.2973
ND3	1	44	44	8	16	0	-0.3333
major	2	21	69	12	10	-0.5333	0.0909
	3	35	59	8	10	-0.2553	-0.1111
ND1	1	129	107	31	49	0.0932	-0.225
minor	2	62	155	43	56	-0.4286	-0.1313
	3	134	130	29	23	0.0152	0.1154
ND5	1	229	199	43	81	0.0701	-0.3065
minor	2	115	293	79	65	-0.4363	0.0972
	3	219	245	48	40	-0.056	0.0909
ND4	1	155	176	42	65	-0.0634	-0.215
minor	2	77	239	56	66	-0.5127	-0.082
	3	188	191	27	32	-0.0079	-0.0847
ND4L	1	36	39	6	11	-0.04	-0.2941
minor	2	18	54	9	11	-0.5	-0.1
	3	30	49	3	10	-0.2405	-0.5385
ND6	1	73	49	13	12	0.1967	0.04
major	2	33	88	21	5	-0.4545	0.6154
	3	64	60	15	8	0.0323	0.3043
CytB	1	122	122	47	60	0	-0.1215
major	2	74	167	73	37	-0.3859	0.3273
	3	146	154	34	17	-0.0267	0.3333
all	1	1368	1291	473	469	0.029	0.0042
	2	1195	1360	622	424	-0.0646	0.1893
	3	1516	1552	310	223	-0.0117	0.1632

PARASITIFORMES *Carios capensis*

		A	T	C	G	ATskew	CGskew
ND2	1	127	124	37	30	0.012	0.1045
major	2	59	168	66	25	-0.4802	0.4505
	3	132	133	44	9	-0.0038	0.6604
CO1	1	155	142	95	121	0.0438	-0.1204
major	2	91	223	122	77	-0.4204	0.2261
	3	229	179	95	10	0.1225	0.8095
CO2	1	79	64	42	40	0.1049	0.0244
major	2	56	95	48	26	-0.2583	0.2973
	3	97	78	42	8	0.1086	0.68
ATP8	1	23	21	8	0	0.0455	1
major	2	12	24	13	3	-0.3333	0.625
	3	22	18	10	2	0.1	0.6667
ATP6	1	84	75	40	24	0.0566	0.25
major	2	43	111	50	19	-0.4416	0.4493
	3	84	92	42	5	-0.0455	0.7872
CO3	1	83	90	45	42	-0.0405	0.0345
major	2	55	107	62	36	-0.321	0.2653
	3	95	104	53	8	-0.0452	0.7377
ND3	1	45	34	21	12	0.1392	0.2727
major	2	24	60	19	9	-0.4286	0.3571
	3	48	41	19	4	0.0787	0.6522
ND5	1	194	202	36	121	-0.0202	-0.5414
minor	2	108	289	63	93	-0.4559	-0.1923
	3	201	267	12	73	-0.141	-0.7176
ND4	1	132	167	45	94	-0.1171	-0.3525
minor	2	72	239	46	81	-0.537	-0.2756
	3	167	198	11	62	-0.0849	-0.6986
ND4L	1	30	33	3	27	-0.0476	-0.8
minor	2	16	52	1	24	-0.5294	-0.92
	3	40	34	1	18	0.0811	-0.8947
ND6	1	69	49	20	5	0.1695	0.6
major	2	22	83	32	6	-0.581	0.6842
	3	68	49	22	4	0.1624	0.6923
CytB	1	124	110	71	55	0.0598	0.127
major	2	69	162	90	39	-0.4026	0.3953
	3	146	130	77	7	0.058	0.8333
ND1	1	96	132	24	74	-0.1579	-0.5102
minor	2	72	169	31	54	-0.4025	-0.2706
	3	107	154	6	59	-0.1801	-0.8154
all	1	1323	1161	695	437	0.0652	0.2279
	2	1180	1301	754	381	-0.0488	0.3286
	3	1574	1339	616	87	0.0807	0.7525

PARASITIFORMES *Ornithodoros moubata*

		A	T	C	G	ATskew	CGskew
ND2	1	129	98	62	31	0.1366	0.3333
major	2	63	166	64	27	-0.4498	0.4066
	3	137	136	43	4	0.0037	0.8298
CO1	1	158	147	91	117	0.0361	-0.125
major	2	93	222	119	79	-0.4095	0.202
	3	212	196	84	21	0.0392	0.6
CO2	1	81	64	43	37	0.1172	0.075
major	2	59	92	48	26	-0.2185	0.2973
	3	93	85	42	5	0.0449	0.7872
ATP8	1	24	14	10	4	0.2632	0.4286
major	2	16	24	8	4	-0.2	0.3333
	3	29	16	7	0	0.2889	1
ATP6	1	95	63	43	22	0.2025	0.3231
major	2	43	107	53	20	-0.4267	0.4521
	3	99	75	46	3	0.1379	0.8776
CO3	1	96	74	43	46	0.1294	-0.0337
major	2	53	110	58	38	-0.3497	0.2083
	3	114	88	48	9	0.1287	0.6842
ND3	1	52	34	14	11	0.2093	0.12
major	2	24	64	14	9	-0.4545	0.2174
	3	44	45	21	1	-0.0112	0.9091
ND5	1	169	197	44	143	-0.0765	-0.5294
minor	2	105	284	73	91	-0.4602	-0.1098
	3	166	272	15	100	-0.242	-0.7391
ND4	1	125	175	35	105	-0.1667	-0.5
minor	2	66	246	45	83	-0.5769	-0.2969
	3	136	187	11	106	-0.1579	-0.812
ND4L	1	34	34	4	21	0	-0.68
minor	2	14	57	2	20	-0.6056	-0.8182
	3	32	41	0	20	-0.1233	-1
ND6	1	73	34	28	10	0.3645	0.4737
major	2	28	74	33	10	-0.451	0.5349
	3	66	48	29	2	0.1579	0.871
CytB	1	130	108	65	58	0.0924	0.0569
major	2	66	161	96	38	-0.4185	0.4328
	3	152	129	73	7	0.0819	0.825
ND1	1	96	128	22	79	-0.1429	-0.5644
minor	2	68	161	31	65	-0.4061	-0.3542
	3	95	153	8	69	-0.2339	-0.7922
all	1	1372	1060	747	441	0.1283	0.2576
	2	1193	1273	752	402	-0.0324	0.3033
	3	1599	1247	688	86	0.1237	0.7778

PARASITIFORMES *Stylochyrus rarior*

		A	T	C	G	ATskew	CGskew
ND2	1	150	101	43	27	0.1952	0.2286
major	2	68	161	53	39	-0.4061	0.1522
	3	144	121	49	7	0.0868	0.75
CO1	1	172	132	88	116	0.1316	-0.1373
major	2	95	215	117	81	-0.3871	0.1818
	3	215	188	89	16	0.067	0.6952
CO2	1	87	64	40	34	0.1523	0.0811
major	2	59	92	45	29	-0.2185	0.2162
	3	89	83	41	12	0.0349	0.5472
ATP8	1	28	18	6	1	0.2174	0.7143
major	2	14	24	13	2	-0.2632	0.7333
	3	24	19	10	0	0.1163	1
ATP6	1	95	68	36	23	0.1656	0.2203
major	2	48	101	49	24	-0.3557	0.3425
	3	91	88	38	5	0.0168	0.7674
CO3	1	87	70	49	49	0.1083	0
major	2	53	110	48	39	-0.3497	0.1034
	3	112	90	46	7	0.1089	0.7358
ND3	1	39	36	22	16	0.04	0.1579
major	2	22	60	19	12	-0.4634	0.2258
	3	43	42	22	6	0.0118	0.5714
ND5	1	165	218	35	138	-0.1384	-0.5954
minor	2	103	283	81	89	-0.4663	-0.0471
	3	180	262	9	105	-0.1855	-0.8421
ND4	1	127	208	23	85	-0.2418	-0.5741
minor	2	96	213	50	84	-0.3786	-0.2537
	3	124	228	24	61	-0.2955	-0.4353
ND4L	1	25	35	7	24	-0.1667	-0.5484
minor	2	15	51	6	19	-0.5455	-0.52
	3	37	41	3	10	-0.0513	-0.5385
ND6	1	71	37	25	16	0.3148	0.2195
major	2	29	75	31	14	-0.4423	0.3778
	3	57	56	32	4	0.0088	0.7778
CytB	1	116	109	75	62	0.0311	0.0949
major	2	71	163	79	49	-0.3932	0.2344
	3	120	135	84	23	-0.0588	0.5701
ND1	1	75	119	23	84	-0.2268	-0.5701
minor	2	60	145	46	50	-0.4146	-0.0417
	3	99	137	11	52	-0.161	-0.6508
all	1	1425	1027	715	432	0.1623	0.2467
	2	1151	1275	696	472	-0.0511	0.1918
	3	1563	1262	639	127	0.1065	0.6684

PARASITIFORMES *Varroa destructor*

		A	T	C	G	ATskew	CGskew
ND2	1	136	118	17	44	0.0709	-0.4426
major	2	70	186	17	42	-0.4531	-0.4237
	3	150	132	8	25	0.0638	-0.5152
CO1	1	166	160	60	129	0.0184	-0.3651
major	2	92	236	98	89	-0.439	0.0481
	3	187	287	9	32	-0.211	-0.561
CO2	1	82	78	26	49	0.025	-0.3067
major	2	68	99	33	35	-0.1856	-0.0294
	3	96	122	2	15	-0.1193	-0.7647
ATP8	1	12	15	4	2	-0.1111	0.3333
major	2	12	17	3	1	-0.1724	0.5
	3	14	16	0	3	-0.0667	-1
ATP6	1	82	87	15	37	-0.0296	-0.4231
major	2	45	115	36	25	-0.4375	0.1803
	3	106	91	4	20	0.0761	-0.6667
CO3	1	89	100	25	46	-0.0582	-0.2958
major	2	60	128	30	42	-0.3617	-0.1667
	3	108	134	5	13	-0.1074	-0.4444
ND3	1	46	43	5	14	0.0337	-0.4737
major	2	22	64	10	12	-0.4884	-0.0909
	3	40	60	1	7	-0.2	-0.75
ND5	1	246	189	70	64	0.131	0.0448
minor	2	135	279	99	56	-0.3478	0.2774
	3	200	321	44	4	-0.2322	0.8333
ND4	1	176	154	54	50	0.0667	0.0385
minor	2	92	237	53	52	-0.4407	0.0095
	3	166	229	36	3	-0.1595	0.8462
ND4L	1	36	30	10	11	0.0909	-0.0476
minor	2	18	54	5	10	-0.5	-0.3333
	3	35	49	3	0	-0.1667	1
ND6	1	65	62	7	12	0.0236	-0.2632
major	2	25	94	15	12	-0.5798	0.1111
	3	71	65	2	8	0.0441	-0.6
CytB	1	120	131	40	70	-0.0438	-0.2727
major	2	78	167	59	57	-0.3633	0.0172
	3	133	188	12	28	-0.1713	-0.4
ND1	1	124	104	40	45	0.0877	-0.0588
minor	2	70	152	49	42	-0.3694	0.0769
	3	104	182	17	10	-0.2727	0.2593
all	1	1275	1376	369	577	-0.0381	-0.2199
	2	1194	1421	461	521	-0.0868	-0.0611
	3	1686	1600	60	251	0.0262	-0.6141

RICINULEI *Pseudocellus pearsii*

		A	T	C	G	ATskew	CGskew
ND2	1	143	108	50	20	0.1394	0.4286
major	2	68	148	74	31	-0.3704	0.4095
	3	161	78	76	6	0.3473	0.8537
CO1	1	159	144	95	118	0.0495	-0.108
major	2	99	213	129	75	-0.3654	0.2647
	3	209	146	145	16	0.1775	0.8012
CO2	1	88	53	49	33	0.2482	0.1951
major	2	61	88	48	26	-0.1812	0.2973
	3	105	59	55	4	0.2805	0.8644
ATP8	1	22	14	14	1	0.2222	0.8667
major	2	15	16	15	5	-0.0323	0.5
	3	29	10	12	0	0.4872	1
ATP6	1	96	56	48	24	0.2632	0.3333
major	2	41	100	58	23	-0.4184	0.4321
	3	132	49	40	4	0.4586	0.8182
CO3	1	75	88	51	46	-0.0798	0.0515
major	2	54	109	59	38	-0.3374	0.2165
	3	120	57	75	7	0.3559	0.8293
ND3	1	43	40	18	10	0.0361	0.2857
major	2	23	53	26	9	-0.3947	0.4857
	3	52	30	27	2	0.2683	0.8621
ND5	1	139	231	23	169	-0.2486	-0.7604
minor	2	86	294	66	116	-0.5474	-0.2747
	3	101	321	1	139	-0.5213	-0.9857
ND4	1	107	186	34	112	-0.2696	-0.5342
minor	2	70	232	45	92	-0.5364	-0.3431
	3	93	202	9	135	-0.3695	-0.875
ND4L	1	19	43	3	26	-0.3871	-0.7931
minor	2	14	55	2	20	-0.5942	-0.8182
	3	19	45	0	27	-0.4063	-1
ND6	1	70	34	29	10	0.3462	0.4872
major	2	30	70	30	13	-0.4	0.3953
	3	72	27	41	3	0.4545	0.8636
CytB	1	133	100	78	51	0.1416	0.2093
major	2	73	162	86	41	-0.3787	0.3543
	3	173	61	124	4	0.4786	0.9375
ND1	1	57	127	22	89	-0.3804	-0.6036
minor	2	54	142	35	64	-0.449	-0.2929
	3	40	169	4	82	-0.6172	-0.907
all	1	1416	959	828	395	0.1924	0.354
	2	1187	1183	817	409	0.0017	0.3328
	3	1790	770	978	60	0.3984	0.8844

THELYPHONIDA *Mastigoproctus giganteus*

		A	T	C	G	ATskew	CGskew
ND2	1	124	130	45	29	-0.0236	0.2162
major	2	55	162	77	34	-0.4931	0.3874
	3	135	138	42	13	-0.011	0.5273
CO1	1	139	138	98	127	0.0036	-0.1289
major	2	85	214	124	78	-0.4314	0.2277
	3	202	206	70	24	-0.0098	0.4894
CO2	1	76	51	55	42	0.1969	0.134
major	2	59	93	49	24	-0.2237	0.3425
	3	71	94	49	10	-0.1394	0.661
ATP8	1	19	19	9	4	0	0.3846
major	2	11	21	14	5	-0.3125	0.4737
	3	23	15	10	3	0.2105	0.5385
ATP6	1	78	73	35	38	0.0331	-0.0411
major	2	39	107	61	17	-0.4658	0.5641
	3	92	80	41	11	0.0698	0.5769
CO3	1	58	90	52	61	-0.2162	-0.0796
major	2	46	106	63	46	-0.3947	0.156
	3	99	96	53	13	0.0154	0.6061
ND3	1	46	34	16	18	0.15	-0.0588
major	2	20	54	27	13	-0.4595	0.35
	3	43	38	28	5	0.0617	0.697
ND5	1	176	196	50	135	-0.0538	-0.4595
minor	2	103	280	74	100	-0.4621	-0.1494
	3	210	242	20	85	-0.0708	-0.619
ND4	1	143	149	44	105	-0.0205	-0.4094
minor	2	67	229	64	81	-0.5473	-0.1172
	3	171	193	16	61	-0.0604	-0.5844
ND4L	1	34	29	8	22	0.0794	-0.4667
minor	2	10	55	5	23	-0.6923	-0.6429
	3	44	34	3	12	0.1282	-0.6
ND6	1	51	53	26	18	-0.0192	0.1818
major	2	20	80	37	11	-0.6	0.5417
	3	53	50	30	8	0.0364	0.5789
CytB	1	123	110	69	62	0.0558	0.0534
major	2	66	165	91	42	-0.4286	0.3684
	3	140	134	70	20	0.0219	0.5556
ND1	1	73	105	29	88	-0.1798	-0.5043
minor	2	58	141	38	58	-0.4171	-0.2083
	3	113	118	15	49	-0.0216	-0.5313
all	1	1193	1124	755	530	0.0298	0.1751
	2	1106	1240	805	451	-0.0571	0.2818
	3	1449	1392	600	161	0.0201	0.5769

AMBLYPYGI *Phrynus* sp.

		A	T	C	G	ATskew	CGskew
ND2	1	118	110	64	34	0.0351	0.3061
major	2	58	167	79	22	-0.4844	0.5644
	3	107	145	60	14	-0.1508	0.6216
CO1	1	139	130	104	139	0.0335	-0.144
major	2	86	212	135	79	-0.4228	0.2617
	3	186	199	109	18	-0.0338	0.7165
CO2	1	78	49	57	40	0.2283	0.1753
major	2	60	88	48	28	-0.1892	0.2632
	3	69	99	51	5	-0.1786	0.8214
ATP8	1	19	24	8	1	-0.1163	0.7778
major	2	12	18	15	7	-0.2	0.3636
	3	20	20	12	0	0	1
ATP6	1	74	63	56	30	0.0803	0.3023
major	2	35	108	59	21	-0.5105	0.475
	3	71	101	45	6	-0.1744	0.7647
CO3	1	80	75	58	49	0.0323	0.0841
major	2	58	103	63	38	-0.2795	0.2475
	3	90	99	67	6	-0.0476	0.8356
ND3	1	31	38	34	12	-0.1014	0.4783
major	2	17	64	24	10	-0.5802	0.4118
	3	34	51	26	4	-0.2	0.7333
ND5	1	184	181	28	166	0.0082	-0.7113
minor	2	94	279	69	117	-0.496	-0.2581
	3	201	223	14	121	-0.0519	-0.7926
ND4	1	126	162	34	125	-0.125	-0.5723
minor	2	69	222	49	107	-0.5258	-0.3718
	3	173	145	15	114	0.0881	-0.7674
ND4L	1	28	27	6	34	0.0182	-0.7
minor	2	8	55	3	29	-0.746	-0.8125
	3	27	37	3	28	-0.1563	-0.8065
ND6	1	54	44	36	18	0.102	0.3333
major	2	24	82	38	8	-0.5472	0.6522
	3	50	60	39	3	-0.0909	0.8571
CytB	1	112	103	93	54	0.0419	0.2653
major	2	73	161	82	46	-0.3761	0.2813
	3	98	147	111	6	-0.2	0.8974
ND1	1	76	107	18	102	-0.1694	-0.7
minor	2	50	153	33	67	-0.5074	-0.34
	3	84	116	6	97	-0.16	-0.8835
all	1	1182	1050	937	463	0.0591	0.3386
	2	1132	1224	863	413	-0.039	0.3527
	3	1246	1406	880	100	-0.0603	0.7959

AMBLYPYGI *Damon diadema*

		A	T	C	G	ATskew	CGskew
ND2	1	123	92	71	35	0.1442	0.3396
major	2	56	152	90	23	-0.4615	0.5929
	3	121	98	87	15	0.105	0.7059
CO1	1	142	130	102	139	0.0441	-0.1535
major	2	88	210	140	75	-0.4094	0.3023
	3	189	154	144	26	0.102	0.6941
CO2	1	68	50	61	46	0.1525	0.1402
major	2	54	86	56	29	-0.2286	0.3176
	3	92	61	65	7	0.2026	0.8056
ATP8	1	19	18	13	2	0.027	0.7333
major	2	11	23	15	3	-0.3529	0.6667
	3	23	11	17	1	0.3529	0.8889
ATP6	1	78	60	52	33	0.1304	0.2235
major	2	36	104	64	19	-0.4857	0.5422
	3	86	59	65	13	0.1862	0.6667
CO3	1	78	72	60	51	0.04	0.0811
major	2	51	104	65	41	-0.3419	0.2264
	3	99	72	80	10	0.1579	0.7778
ND3	1	40	34	24	16	0.0811	0.2
major	2	19	61	23	11	-0.525	0.3529
	3	50	29	34	1	0.2658	0.9429
ND5	1	141	188	32	206	-0.1429	-0.7311
minor	2	76	268	71	152	-0.5581	-0.3632
	3	120	251	22	174	-0.3531	-0.7755
ND4	1	92	175	31	151	-0.3109	-0.6593
minor	2	60	219	39	131	-0.5699	-0.5412
	3	90	192	22	145	-0.3617	-0.7365
ND4L	1	21	35	7	41	-0.25	-0.7083
minor	2	13	54	7	30	-0.6119	-0.6216
	3	15	49	5	35	-0.5313	-0.75
ND6	1	61	30	37	22	0.3407	0.2542
major	2	16	74	53	7	-0.6444	0.7667
	3	58	30	58	4	0.3182	0.871
CytB	1	107	104	90	61	0.0142	0.1921
major	2	69	158	91	44	-0.3921	0.3481
	3	124	121	97	20	0.0122	0.6581
ND1	1	65	104	23	112	-0.2308	-0.6593
minor	2	49	155	35	65	-0.5196	-0.3
	3	71	137	5	91	-0.3173	-0.8958
all	1	1218	909	1020	498	0.1453	0.3439
	2	1096	1170	975	404	-0.0327	0.4141
	3	1471	931	1092	151	0.2248	0.757

ARANEAE *Heptathela hangzhouensis*

		A	T	C	G	ATskew	CGskew
ND2	1	117	128	49	27	-0.0449	0.2895
major	2	55	165	72	29	-0.5	0.4257
	3	124	129	50	18	-0.0198	0.4706
CO1	1	152	142	93	124	0.034	-0.1429
major	2	90	220	125	76	-0.4194	0.2438
	3	191	240	49	31	-0.1137	0.225
CO2	1	62	67	46	47	-0.0388	-0.0108
major	2	54	90	52	26	-0.25	0.3333
	3	92	87	30	13	0.0279	0.3953
ATP8	1	16	23	9	2	-0.1795	0.6364
major	2	15	20	12	3	-0.1429	0.6
	3	17	20	10	3	-0.0811	0.5385
ATP6	1	90	67	33	31	0.1465	0.0313
major	2	34	116	54	17	-0.5467	0.5211
	3	91	89	26	15	0.0111	0.2683
CO3	1	70	84	53	51	-0.0909	0.0192
major	2	52	106	60	40	-0.3418	0.2
	3	104	108	30	16	-0.0189	0.3043
ND3	1	42	41	15	12	0.012	0.1111
major	2	17	61	22	10	-0.5641	0.375
	3	47	48	10	5	-0.0105	0.3333
ND5	1	173	195	48	129	-0.0598	-0.4576
minor	2	87	275	86	97	-0.5193	-0.0601
	3	217	222	40	66	-0.0114	-0.2453
ND4	1	134	158	50	91	-0.0822	-0.2908
minor	2	72	228	55	78	-0.52	-0.1729
	3	182	171	22	58	0.0312	-0.45
ND4L	1	28	35	9	22	-0.1111	-0.4194
minor	2	15	51	4	24	-0.5455	-0.7143
	3	47	33	5	9	0.175	-0.2857
ND6	1	60	54	18	12	0.0526	0.2
major	2	22	81	33	8	-0.5728	0.6098
	3	60	63	15	6	-0.0244	0.4286
CytB	1	108	109	74	69	-0.0046	0.035
major	2	68	167	77	48	-0.4213	0.232
	3	139	143	59	19	-0.0142	0.5128
ND1	1	88	121	24	72	-0.1579	-0.5
minor	2	58	155	35	57	-0.4554	-0.2391
	3	124	131	12	38	-0.0275	-0.52
all	1	1226	1138	704	506	0.0372	0.1636
	2	1116	1258	763	437	-0.0598	0.2717
	3	1422	1497	450	205	-0.0257	0.374

ARANEAE *Hypochilus thorelli*

		A	T	C	G	ATskew	CGskew
ND2	1	93	132	23	72	-0.1733	-0.5158
major	2	57	166	37	60	-0.4888	-0.2371
	3	90	139	13	78	-0.214	-0.7143
CO1	1	141	156	66	148	-0.0505	-0.3832
major	2	97	221	106	88	-0.3899	0.0928
	3	120	242	43	107	-0.337	-0.4267
CO2	1	55	73	29	61	-0.1406	-0.3556
major	2	59	95	34	30	-0.2338	0.0625
	3	51	102	14	51	-0.3333	-0.5692
ATP8	1	18	22	4	7	-0.1	-0.2727
major	2	12	25	6	8	-0.3514	-0.1429
	3	17	24	3	7	-0.1707	-0.4
ATP6	1	73	74	28	47	-0.0068	-0.2533
major	2	30	121	35	36	-0.6026	-0.0141
	3	61	108	10	43	-0.2781	-0.6226
CO3	1	62	103	35	62	-0.2485	-0.2784
major	2	45	125	48	44	-0.4706	0.0435
	3	65	121	16	60	-0.3011	-0.5789
ND3	1	33	48	10	24	-0.1852	-0.4118
major	2	21	68	13	13	-0.5281	0
	3	32	42	13	28	-0.1351	-0.3659
ND5	1	213	180	63	88	0.084	-0.1656
minor	2	91	255	149	49	-0.474	0.5051
	3	264	139	117	24	0.3102	0.6596
ND4	1	185	132	63	49	0.1672	0.125
minor	2	88	211	92	38	-0.4114	0.4154
	3	177	125	97	30	0.1722	0.5276
ND4L	1	30	30	16	10	0	0.2308
minor	2	7	47	19	13	-0.7407	0.1875
	3	39	22	18	7	0.2787	0.44
ND6	1	48	59	6	28	-0.1028	-0.6471
major	2	28	84	14	15	-0.5	-0.0345
	3	45	62	10	24	-0.1589	-0.4118
CytB	1	92	137	46	90	-0.1965	-0.3235
major	2	70	176	57	62	-0.4309	-0.042
	3	91	183	24	67	-0.3358	-0.4725
ND1	1	107	102	50	47	0.0239	0.0309
minor	2	58	151	61	36	-0.445	0.2577
	3	122	100	70	14	0.0991	0.6667
all	1	1059	1339	441	731	-0.1168	-0.2474
	2	1083	1325	486	677	-0.1005	-0.1642
	3	958	1625	221	767	-0.2582	-0.5526

ARANEAE *Habronattus oregonensis*

		A	T	C	G	ATskew	CGskew
ND2	1	97	124	24	75	-0.1222	-0.5152
major	2	61	170	26	63	-0.4719	-0.4157
	3	110	147	11	52	-0.144	-0.6508
CO1	1	137	167	57	153	-0.0987	-0.4571
major	2	89	226	112	84	-0.4349	0.1429
	3	186	270	6	51	-0.1842	-0.7895
CO2	1	55	74	26	66	-0.1473	-0.4348
major	2	58	93	34	35	-0.2318	-0.0145
	3	78	112	3	29	-0.1789	-0.8125
ATP8	1	21	13	4	12	0.2353	-0.5
major	2	12	28	3	8	-0.4	-0.4545
	3	24	21	1	5	0.0667	-0.6667
ATP6	1	67	81	21	53	-0.0946	-0.4324
major	2	32	116	37	37	-0.5676	0
	3	96	98	5	22	-0.0103	-0.6296
CO3	1	64	99	36	63	-0.2147	-0.2727
major	2	50	124	42	46	-0.4253	-0.0455
	3	95	136	7	24	-0.1775	-0.5484
ND3	1	32	51	7	24	-0.2289	-0.5484
major	2	26	63	11	14	-0.4157	-0.12
	3	48	56	3	7	-0.0769	-0.4
ND5	1	229	171	73	72	0.145	0.0069
minor	2	101	256	136	52	-0.4342	0.4468
	3	284	168	84	9	0.2566	0.8065
ND4	1	193	117	65	53	0.2452	0.1017
minor	2	73	227	96	32	-0.5133	0.5
	3	205	158	60	5	0.1295	0.8462
ND4L	1	40	34	9	6	0.0811	0.2
minor	2	10	46	21	12	-0.6429	0.2727
	3	55	29	4	1	0.3095	0.6
ND6	1	53	52	4	34	0.0095	-0.7895
major	2	24	83	8	28	-0.5514	-0.5556
	3	70	54	3	16	0.129	-0.6842
CytB	1	102	137	38	86	-0.1464	-0.3871
major	2	72	171	60	60	-0.4074	0
	3	117	192	6	48	-0.2427	-0.7778
ND1	1	116	104	45	42	0.0545	0.0345
minor	2	67	145	55	40	-0.3679	0.1579
	3	139	121	45	2	0.0692	0.9149
all	1	1054	1376	390	758	-0.1325	-0.3206
	2	1098	1325	469	683	-0.0937	-0.1858
	3	1300	1769	62	447	-0.1528	-0.7564

ARANEAE *Nephila clavata*

		A	T	C	G	ATskew	CGskew
ND2	1	120	126	17	53	-0.0244	-0.5143
major	2	53	174	31	58	-0.533	-0.3034
	3	138	127	7	44	0.0415	-0.7255
CO1	1	132	175	61	144	-0.1401	-0.4049
major	2	91	224	110	87	-0.4222	0.1168
	3	194	254	8	56	-0.1339	-0.75
CO2	1	68	70	34	49	-0.0145	-0.1807
major	2	55	95	37	34	-0.2667	0.0423
	3	79	116	3	23	-0.1897	-0.7692
ATP8	1	24	20	3	6	0.0909	-0.3333
major	2	14	26	5	8	-0.3	-0.2308
	3	30	22	0	1	0.1538	-1
ATP6	1	59	83	26	53	-0.169	-0.3418
major	2	35	113	33	40	-0.527	-0.0959
	3	86	107	7	21	-0.1088	-0.5
CO3	1	66	98	29	68	-0.1951	-0.4021
major	2	47	122	39	53	-0.4438	-0.1522
	3	96	127	7	31	-0.139	-0.6316
ND3	1	34	48	7	22	-0.1707	-0.5172
major	2	20	66	11	14	-0.5349	-0.12
	3	37	58	5	11	-0.2211	-0.375
ND5	1	220	187	65	73	0.0811	-0.058
minor	2	92	268	138	47	-0.4889	0.4919
	3	243	242	50	10	0.0021	0.6667
ND4	1	167	150	60	48	0.0536	0.1111
minor	2	76	226	91	32	-0.4967	0.4797
	3	181	194	45	5	-0.0347	0.8
ND4L	1	34	38	12	6	-0.0556	0.3333
minor	2	12	51	15	12	-0.619	0.1111
	3	43	39	5	3	0.0488	0.25
ND6	1	57	46	5	34	0.1068	-0.7436
major	2	21	84	8	29	-0.6	-0.5676
	3	70	47	3	22	0.1966	-0.76
CytB	1	106	132	47	78	-0.1092	-0.248
major	2	67	173	63	60	-0.4417	0.0244
	3	124	195	10	34	-0.2226	-0.5455
ND1	1	106	111	40	45	-0.023	-0.0588
minor	2	59	148	55	40	-0.43	0.1579
	3	118	147	28	9	-0.1094	0.5135
all	1	1152	1325	401	684	-0.0698	-0.2608
	2	1096	1316	468	682	-0.0912	-0.1861
	3	1476	1638	77	371	-0.052	-0.6563

ARANEAE *Ornithoctonus huwena*

		A	T	C	G	ATskew	CGskew
ND2	1	108	105	17	81	0.0141	-0.6531
major	2	52	166	30	63	-0.5229	-0.3548
	3	117	123	10	61	-0.025	-0.7183
CO1	1	124	167	65	156	-0.1478	-0.4118
major	2	89	230	106	87	-0.442	0.0984
	3	167	243	17	85	-0.1854	-0.6667
CO2	1	63	64	29	67	-0.0079	-0.3958
major	2	59	91	35	38	-0.2133	-0.0411
	3	78	103	1	41	-0.1381	-0.9524
ATP8	1	23	15	4	9	0.2105	-0.3846
major	2	14	23	6	8	-0.2432	-0.1429
	3	23	16	0	12	0.1795	-1
ATP6	1	70	79	19	55	-0.0604	-0.4865
major	2	31	115	40	37	-0.5753	0.039
	3	75	106	9	33	-0.1713	-0.5714
CO3	1	52	96	34	79	-0.2973	-0.3982
major	2	46	122	44	49	-0.4524	-0.0538
	3	74	115	11	61	-0.2169	-0.6944
ND3	1	24	44	9	30	-0.2941	-0.5385
major	2	22	59	11	15	-0.4568	-0.1538
	3	24	51	3	29	-0.36	-0.8125
ND5	1	208	179	88	71	0.0749	0.1069
minor	2	97	253	147	49	-0.4457	0.5
	3	241	182	110	13	0.1395	0.7886
ND4	1	151	148	74	57	0.01	0.1298
minor	2	72	217	108	33	-0.5017	0.5319
	3	186	147	91	6	0.1171	0.8763
ND4L	1	30	34	18	10	-0.0625	0.2857
minor	2	12	45	22	13	-0.5789	0.2571
	3	42	33	13	4	0.12	0.5294
ND6	1	61	48	5	29	0.1193	-0.7059
major	2	23	80	13	27	-0.5534	-0.35
	3	51	59	1	32	-0.0727	-0.9394
CytB	1	101	130	45	88	-0.1255	-0.3233
major	2	65	172	62	65	-0.4515	-0.0236
	3	94	188	9	73	-0.3333	-0.7805
ND1	1	99	104	41	60	-0.0246	-0.1881
minor	2	54	144	70	36	-0.4545	0.3208
	3	134	108	58	4	0.1074	0.871
all	1	1091	1236	425	815	-0.0623	-0.3145
	2	1060	1293	478	736	-0.099	-0.2125
	3	1173	1607	88	699	-0.1561	-0.7764

ARANEAE *Calisoga longitarsis*

		A	T	C	G	ATskew	CGskew
ND2	1	68	119	25	99	-0.2727	-0.5968
major	2	43	164	30	74	-0.5845	-0.4231
	3	52	145	11	103	-0.4721	-0.807
CO1	1	119	168	66	159	-0.1707	-0.4133
major	2	79	229	106	98	-0.487	0.0392
	3	91	234	23	162	-0.44	-0.7514
CO2	1	51	58	35	78	-0.0642	-0.3805
major	2	60	90	35	37	-0.2	-0.0278
	3	43	99	17	63	-0.3944	-0.575
ATP8	1	12	15	7	16	-0.1111	-0.3913
major	2	10	25	6	9	-0.4286	-0.2
	3	11	21	0	18	-0.3125	-1
ATP6	1	58	74	25	65	-0.1212	-0.4444
major	2	34	118	34	36	-0.5526	-0.0286
	3	40	110	5	67	-0.4667	-0.8611
CO3	1	50	92	31	88	-0.2958	-0.479
major	2	39	128	43	51	-0.5329	-0.0851
	3	56	112	13	80	-0.3333	-0.7204
ND3	1	24	46	9	33	-0.3143	-0.5714
major	2	23	59	15	15	-0.439	0
	3	21	51	9	31	-0.4167	-0.55
ND5	1	189	156	112	90	0.0957	0.1089
minor	2	94	241	155	57	-0.4388	0.4623
	3	204	148	167	28	0.1591	0.7128
ND4	1	139	134	88	68	0.0183	0.1282
minor	2	69	208	120	32	-0.5018	0.5789
	3	161	121	113	34	0.1418	0.5374
ND4L	1	36	25	18	7	0.1803	0.44
minor	2	6	44	22	14	-0.76	0.2222
	3	25	23	31	7	0.0417	0.6316
ND6	1	44	46	12	41	-0.0222	-0.5472
major	2	17	81	16	29	-0.6531	-0.2889
	3	35	52	8	48	-0.1954	-0.7143
CytB	1	87	121	48	108	-0.1635	-0.3846
major	2	64	176	62	62	-0.4667	0
	3	94	157	29	84	-0.251	-0.4867
ND1	1	94	103	48	60	-0.0457	-0.1111
minor	2	52	152	61	40	-0.4902	0.2079
	3	117	89	83	16	0.1359	0.6768
all	1	931	1197	483	953	-0.125	-0.3273
	2	1014	1291	490	769	-0.1202	-0.2216
	3	824	1488	200	1050	-0.2872	-0.68

SCORPIONES *Uroctonus mordax*

		A	T	C	G	ATskew	CGskew
ND2	1	91	133	24	75	-0.1875	-0.5152
major	2	47	167	60	49	-0.5607	0.1009
	3	126	141	25	31	-0.0562	-0.1071
CO1	1	133	161	73	146	-0.0952	-0.3333
major	2	84	219	120	90	-0.4455	0.1429
	3	146	304	12	51	-0.3511	-0.619
CO2	1	57	67	34	65	-0.0806	-0.3131
major	2	55	94	39	35	-0.2617	0.0541
	3	63	129	0	31	-0.3438	-1
ATP8	1	14	24	3	11	-0.2632	-0.5714
major	2	11	30	4	7	-0.4634	-0.2727
	3	22	24	1	5	-0.0435	-0.6667
ATP6	1	65	81	22	54	-0.1096	-0.4211
major	2	30	116	42	34	-0.589	0.1053
	3	75	122	3	22	-0.2386	-0.76
CO3	1	67	99	30	64	-0.1928	-0.3617
major	2	49	111	44	56	-0.3875	-0.12
	3	85	151	4	20	-0.2797	-0.6667
ND3	1	25	55	9	25	-0.375	-0.4706
major	2	22	65	13	14	-0.4943	-0.037
	3	39	55	3	17	-0.1702	-0.7
ND5	1	230	170	72	90	0.15	-0.1111
minor	2	107	274	125	56	-0.4383	0.3812
	3	282	215	59	6	0.1348	0.8154
ND4	1	163	152	59	69	0.0349	-0.0781
minor	2	74	220	88	61	-0.4966	0.1812
	3	225	170	37	11	0.1392	0.5417
ND4L	1	43	29	12	15	0.1944	-0.1111
minor	2	16	55	13	15	-0.5493	-0.0714
	3	51	40	7	1	0.1209	0.75
ND6	1	48	58	10	29	-0.0943	-0.4872
major	2	22	77	24	22	-0.5556	0.0435
	3	65	76	0	4	-0.078	-1
CytB	1	87	141	51	83	-0.2368	-0.2388
major	2	73	167	69	53	-0.3917	0.1311
	3	129	203	8	22	-0.2229	-0.4667
ND1	1	90	121	28	68	-0.1469	-0.4167
minor	2	59	150	50	48	-0.4354	0.0204
	3	133	140	21	13	-0.0256	0.2353
all	1	1059	1345	498	723	-0.119	-0.1843
	2	1092	1302	595	636	-0.0877	-0.0333
	3	1315	1896	87	327	-0.1809	-0.5797

SCORPIONES *Buthus occitanus*

		A	T	C	G	ATskew	CGskew
ND2	1	68	138	27	90	-0.3398	-0.5385
major	2	44	169	45	65	-0.5869	-0.1818
	3	72	135	11	105	-0.3043	-0.8103
CO1	1	126	170	63	152	-0.1486	-0.414
major	2	86	217	118	90	-0.4323	0.1346
	3	102	259	15	134	-0.4349	-0.7987
CO2	1	47	81	29	67	-0.2656	-0.3958
major	2	45	96	43	39	-0.3617	0.0488
	3	38	114	4	68	-0.5	-0.8889
ATP8	1	9	20	6	17	-0.3793	-0.4783
major	2	12	26	3	11	-0.3684	-0.5714
	3	18	21	0	13	-0.0769	-1
ATP6	1	54	88	19	61	-0.2394	-0.525
major	2	34	118	39	31	-0.5526	0.1143
	3	54	105	3	60	-0.3208	-0.9048
CO3	1	51	104	28	77	-0.3419	-0.4667
major	2	51	115	41	53	-0.3855	-0.1277
	3	62	124	11	63	-0.3333	-0.7027
ND3	1	28	42	8	36	-0.2	-0.6364
major	2	19	61	12	22	-0.525	-0.2941
	3	37	45	5	27	-0.0976	-0.6875
ND5	1	174	178	123	84	-0.0114	0.1884
minor	2	97	258	149	55	-0.4535	0.4608
	3	201	203	141	14	-0.005	0.8194
ND4	1	128	150	102	58	-0.0791	0.275
minor	2	63	216	105	54	-0.5484	0.3208
	3	150	169	109	10	-0.0596	0.8319
ND4L	1	34	36	13	13	-0.0286	0
minor	2	15	51	16	14	-0.5455	0.0667
	3	34	42	14	6	-0.1053	0.4
ND6	1	25	64	20	34	-0.4382	-0.2593
major	2	19	86	18	20	-0.6381	-0.0526
	3	50	67	12	15	-0.1453	-0.1111
CytB	1	86	131	64	80	-0.2074	-0.1111
major	2	67	164	72	58	-0.4199	0.1077
	3	99	204	17	41	-0.3465	-0.4138
ND1	1	74	126	38	66	-0.26	-0.2692
minor	2	54	154	54	42	-0.4808	0.125
	3	93	159	22	30	-0.2619	-0.1538
all	1	984	1248	485	890	-0.1183	-0.2945
	2	1056	1281	556	713	-0.0963	-0.1237
	3	1105	1552	138	812	-0.1682	-0.7095

SCORPIONES *Centruroides limpidus*

		A	T	C	G	ATskew	CGskew
ND2	1	46	142	35	97	-0.5106	-0.4697
major	2	36	169	51	64	-0.6488	-0.113
	3	36	182	11	91	-0.6697	-0.7843
CO1	1	122	156	72	161	-0.1223	-0.382
major	2	85	213	120	93	-0.4295	0.1268
	3	48	305	23	135	-0.728	-0.7089
CO2	1	50	71	33	70	-0.1736	-0.3592
major	2	49	96	39	40	-0.3241	-0.0127
	3	23	130	5	66	-0.6993	-0.8592
ATP8	1	5	24	5	18	-0.6552	-0.5652
major	2	10	22	8	12	-0.375	-0.2
	3	7	31	0	14	-0.6316	-1
ATP6	1	48	74	31	69	-0.2131	-0.38
major	2	25	120	40	37	-0.6552	0.039
	3	29	123	2	68	-0.6184	-0.9429
CO3	1	45	100	38	77	-0.3793	-0.3391
major	2	46	111	38	65	-0.414	-0.2621
	3	31	146	13	70	-0.6497	-0.6867
ND3	1	14	47	10	42	-0.541	-0.6154
major	2	19	62	10	22	-0.5309	-0.375
	3	14	55	5	39	-0.5942	-0.7727
ND5	1	230	131	119	79	0.2742	0.202
minor	2	93	244	164	58	-0.4481	0.4775
	3	235	115	185	24	0.3429	0.7703
ND4	1	140	122	121	57	0.0687	0.3596
minor	2	59	208	117	56	-0.5581	0.3526
	3	165	109	142	24	0.2044	0.7108
ND4L	1	37	18	24	17	0.3455	0.1707
minor	2	12	47	23	14	-0.5932	0.2432
	3	42	21	25	8	0.3333	0.5152
ND6	1	19	62	18	45	-0.5309	-0.4286
major	2	18	84	23	19	-0.6471	0.0952
	3	33	74	11	26	-0.3832	-0.4054
CytB	1	72	128	72	89	-0.28	-0.1056
major	2	62	166	70	63	-0.4561	0.0526
	3	81	194	31	55	-0.4109	-0.2791
ND1	1	82	113	40	71	-0.159	-0.2793
minor	2	57	147	55	47	-0.4412	0.0784
	3	88	134	45	39	-0.2072	0.0714
all	1	805	1293	538	972	-0.2326	-0.2874
	2	996	1264	574	774	-0.1186	-0.1484
	3	681	1770	196	961	-0.4443	-0.6612

OPILIONES *Phalangium opilio*

		A	T	C	G	ATskew	CGskew
ND2	1	143	100	42	37	0.177	0.0633
major	2	58	153	74	37	-0.4502	0.3333
	3	138	118	40	26	0.0781	0.2121
CO1	1	157	137	89	130	0.068	-0.1872
major	2	94	216	128	75	-0.3935	0.2611
	3	215	198	72	27	0.0412	0.4545
CO2	1	86	59	36	47	0.1862	-0.1325
major	2	61	86	56	25	-0.1701	0.3827
	3	98	79	42	9	0.1073	0.6471
ATP8	1	26	14	12	1	0.3	0.8462
major	2	17	20	10	6	-0.0811	0.25
	3	29	17	6	1	0.2609	0.7143
ATP6	1	97	61	40	27	0.2278	0.194
major	2	46	105	53	21	-0.3907	0.4324
	3	98	78	36	13	0.1136	0.4694
CO3	1	80	68	58	54	0.0811	0.0357
major	2	54	105	59	42	-0.3208	0.1683
	3	101	99	43	17	0.01	0.4333
ND3	1	38	36	20	19	0.027	0.0256
major	2	22	57	23	11	-0.443	0.3529
	3	51	39	16	7	0.1333	0.3913
ND5	1	140	232	48	141	-0.2473	-0.4921
minor	2	90	288	76	107	-0.5238	-0.1694
	3	180	289	23	69	-0.2324	-0.5
ND4	1	101	180	59	101	-0.2811	-0.2625
minor	2	71	222	64	84	-0.5154	-0.1351
	3	146	205	27	63	-0.1681	-0.4
ND4L	1	20	41	9	27	-0.3443	-0.5
minor	2	15	56	4	22	-0.5775	-0.6923
	3	40	37	2	18	0.039	-0.8
ND6	1	69	42	20	19	0.2432	0.0256
major	2	26	80	32	12	-0.5094	0.4545
	3	64	53	23	10	0.094	0.3939
CytB	1	109	109	70	73	0	-0.021
major	2	75	159	80	47	-0.359	0.2598
	3	133	132	73	22	0.0038	0.5368
ND1	1	63	126	30	81	-0.3333	-0.4595
minor	2	54	150	41	55	-0.4706	-0.1458
	3	96	148	17	39	-0.2131	-0.3929
all	1	1384	950	737	553	0.1859	0.1426
	2	1169	1211	783	461	-0.0176	0.2588
	3	1606	1275	540	201	0.1149	0.4575

XIPHOSURA *Limulus polyphemus*

		A	T	C	G	ATskew	CGskew
ND2	1	128	108	70	33	0.0847	0.3592
major	2	55	158	96	30	-0.4836	0.5238
	3	147	100	86	6	0.1903	0.8696
CO1	1	142	122	108	140	0.0758	-0.129
major	2	92	215	127	78	-0.4007	0.239
	3	222	161	117	12	0.1593	0.814
CO2	1	81	54	44	49	0.2	-0.0538
major	2	56	89	55	28	-0.2276	0.3253
	3	91	68	64	5	0.1447	0.8551
ATP8	1	24	15	10	3	0.2308	0.5385
major	2	12	21	16	3	-0.2727	0.6842
	3	26	15	11	0	0.2683	1
ATP6	1	84	52	57	32	0.2353	0.2809
major	2	37	104	64	20	-0.4752	0.5238
	3	94	72	56	3	0.1325	0.8983
CO3	1	70	76	62	53	-0.0411	0.0783
major	2	49	103	67	42	-0.3553	0.2294
	3	104	78	73	6	0.1429	0.8481
ND3	1	40	39	22	14	0.0127	0.2222
major	2	21	55	29	10	-0.4474	0.4872
	3	54	31	29	1	0.2706	0.9333
ND5	1	142	234	46	148	-0.2447	-0.5258
minor	2	95	275	91	109	-0.4865	-0.09
	3	171	280	18	101	-0.2417	-0.6975
ND4	1	89	199	43	115	-0.3819	-0.4557
minor	2	67	218	61	100	-0.5298	-0.2422
	3	129	209	6	102	-0.2367	-0.8889
ND4L	1	27	38	10	25	-0.1692	-0.4286
minor	2	18	55	4	23	-0.5068	-0.7037
	3	38	36	2	24	0.027	-0.8462
ND6	1	69	40	30	15	0.2661	0.3333
major	2	25	81	41	7	-0.5283	0.7083
	3	66	41	43	4	0.2336	0.8298
CytB	1	111	107	86	60	0.0183	0.1781
major	2	74	156	88	46	-0.3565	0.3134
	3	135	115	107	7	0.08	0.8772
ND1	1	67	123	27	94	-0.2947	-0.5537
minor	2	49	146	46	70	-0.4974	-0.2069
	3	73	160	4	74	-0.3734	-0.8974
all	1	1343	938	871	525	0.1776	0.2479
	2	1115	1211	885	466	-0.0413	0.3101
	3	1624	1092	887	74	0.1959	0.846

XIPHOSURA *Tachypleus tridentatus*

		A	T	C	G	ATskew	CGskew
ND2	1	132	138	42	28	-0.0222	0.2
major	2	63	164	83	30	-0.4449	0.469
	3	147	155	33	5	-0.0265	0.7368
CO1	1	147	136	94	135	0.0389	-0.179
major	2	93	210	131	78	-0.3861	0.2536
	3	228	214	66	4	0.0317	0.8857
CO2	1	87	53	44	44	0.2429	0
major	2	57	89	56	26	-0.2192	0.3659
	3	88	106	31	3	-0.0928	0.8235
ATP8	1	21	21	9	1	0	0.8
major	2	10	26	11	5	-0.4444	0.375
	3	26	22	3	1	0.0833	0.5
ATP6	1	90	76	31	28	0.0843	0.0508
major	2	37	108	60	20	-0.4897	0.5
	3	104	93	25	3	0.0558	0.7857
CO3	1	73	90	46	52	-0.1043	-0.0612
major	2	50	105	63	43	-0.3548	0.1887
	3	110	115	33	3	-0.0222	0.8333
ND3	1	37	44	19	15	-0.0864	0.1176
major	2	19	61	24	11	-0.525	0.3714
	3	41	58	14	2	-0.1717	0.75
ND5	1	162	242	42	125	-0.198	-0.497
minor	2	101	276	88	106	-0.4642	-0.0928
	3	226	294	5	46	-0.1308	-0.8039
ND4	1	122	197	36	91	-0.2351	-0.4331
minor	2	68	226	56	96	-0.5374	-0.2632
	3	187	215	6	38	-0.0697	-0.7273
ND4L	1	33	39	7	21	-0.0833	-0.5
minor	2	18	58	3	21	-0.5263	-0.75
	3	47	34	2	17	0.1605	-0.7895
ND6	1	66	55	21	12	0.0909	0.2727
major	2	23	84	40	7	-0.5701	0.7021
	3	79	52	22	1	0.2061	0.913
CytB	1	109	129	68	58	-0.084	0.0794
major	2	76	163	78	47	-0.364	0.248
	3	143	163	55	3	-0.0654	0.8966
ND1	1	85	125	24	78	-0.1905	-0.5294
minor	2	57	147	43	65	-0.4412	-0.2037
	3	132	137	7	36	-0.0186	-0.6744
all	1	1365	1144	689	482	0.0881	0.1768
	2	1135	1254	834	457	-0.0498	0.292
	3	1646	1570	419	45	0.0236	0.806

SOLIFUGAE *Nothopuga* sp.

		A	T	C	G	ATskew	CGskew
ND2	1	143	105	42	34	0.1532	0.1053
major	2	64	155	78	27	-0.4155	0.4857
	3	146	102	57	19	0.1774	0.5
CO1	1	152	136	92	133	0.0556	-0.1822
major	2	95	214	126	78	-0.3851	0.2353
	3	209	174	102	28	0.0914	0.5692
CO2	1	79	56	43	45	0.1704	-0.0227
major	2	56	88	53	26	-0.2222	0.3418
	3	102	78	34	9	0.1333	0.5814
ATP8	1	27	15	8	3	0.2857	0.4545
major	2	15	26	9	3	-0.2683	0.5
	3	25	10	14	4	0.4286	0.5556
ATP6	1	88	56	44	38	0.2222	0.0732
major	2	40	113	59	14	-0.4771	0.6164
	3	96	77	47	6	0.1098	0.7736
CO3	1	86	80	40	54	0.0361	-0.1489
major	2	53	101	68	38	-0.3117	0.283
	3	103	89	55	13	0.0729	0.6176
ND3	1	44	30	25	16	0.1892	0.2195
major	2	19	58	28	10	-0.5065	0.4737
	3	43	39	25	8	0.0488	0.5152
ND5	1	160	215	46	141	-0.1467	-0.508
minor	2	97	270	72	123	-0.4714	-0.2615
	3	136	290	23	113	-0.3615	-0.6618
ND4	1	131	164	36	114	-0.1119	-0.52
minor	2	72	230	42	101	-0.5232	-0.4126
	3	139	194	17	95	-0.1652	-0.6964
ND4L	1	17	48	5	28	-0.4769	-0.697
minor	2	19	52	7	20	-0.4648	-0.4815
	3	24	48	1	25	-0.3333	-0.9231
ND6	1	66	33	29	17	0.3333	0.2609
major	2	26	79	33	7	-0.5048	0.65
	3	61	45	36	3	0.1509	0.8462
CytB	1	106	106	81	71	0	0.0658
major	2	71	160	89	44	-0.3853	0.3383
	3	141	116	88	19	0.0973	0.6449
ND1	1	72	121	24	89	-0.2539	-0.5752
minor	2	62	149	40	55	-0.4123	-0.1579
	3	71	154	6	75	-0.3689	-0.8519
all	1	1339	997	776	522	0.1464	0.1957
	2	1140	1244	842	408	-0.0436	0.3472
	3	1612	1100	766	156	0.1888	0.6616

SOLIFUGAE *Eremobates palpisetulosus* group

		A	T	C	G	ATskew	CGskew
ND2	1	149	87	54	39	0.2627	0.1613
major	2	57	163	79	30	-0.4818	0.4495
	3	164	103	54	8	0.2285	0.7419
CO1	1	149	142	89	132	0.0241	-0.1946
major	2	93	210	129	80	-0.3861	0.2344
	3	217	190	93	12	0.0663	0.7714
CO2	1	72	62	45	47	0.0746	-0.0217
major	2	56	90	55	25	-0.2329	0.375
	3	95	88	37	6	0.0383	0.7209
ATP8	1	21	15	15	1	0.1667	0.875
major	2	12	22	14	4	-0.2941	0.5556
	3	30	13	7	2	0.3953	0.5556
ATP6	1	88	48	50	39	0.2941	0.1236
major	2	38	107	62	18	-0.4759	0.55
	3	106	77	38	4	0.1585	0.8095
CO3	1	83	79	46	53	0.0247	-0.0707
major	2	55	106	60	40	-0.3168	0.2
	3	117	86	56	2	0.1527	0.931
ND3	1	44	35	21	16	0.1139	0.1351
major	2	22	60	25	9	-0.4634	0.4706
	3	54	31	29	2	0.2706	0.871
ND5	1	164	207	35	157	-0.1159	-0.6354
minor	2	90	266	81	126	-0.4944	-0.2174
	3	195	255	8	105	-0.1333	-0.8584
ND4	1	131	160	34	115	-0.0997	-0.5436
minor	2	78	214	45	103	-0.4658	-0.3919
	3	139	206	10	85	-0.1942	-0.7895
ND4L	1	25	42	6	25	-0.2537	-0.6129
minor	2	17	53	6	22	-0.5143	-0.5714
	3	34	41	2	21	-0.0933	-0.8261
ND6	1	68	34	31	15	0.3333	0.3478
major	2	27	78	36	7	-0.4857	0.6744
	3	75	48	25	0	0.2195	1
CytB	1	112	98	81	73	0.0667	0.0519
major	2	71	156	92	45	-0.3744	0.3431
	3	165	94	92	12	0.2741	0.7692
ND1	1	65	113	25	104	-0.2697	-0.6124
minor	2	60	143	40	63	-0.4089	-0.2233
	3	91	136	8	71	-0.1982	-0.7975
all	1	1308	985	833	515	0.1409	0.2359
	2	1107	1237	866	430	-0.0555	0.3364
	3	1661	1189	713	76	0.1656	0.8074
