# Supplementary figures and images for: Pseudoscorpion mitochondria show rearranged genes and genome-wide reductions of RNA gene sizes and inferred structures, yet typical nucleotide composition bias
Source: BMC Evol Biol. 2012 Mar 12;12:31. doi: 10.1186/1471-2148-12-31 (PMC3325882; doi:10.1186/1471-2148-12-31)

### Skew at 3rd position of codon, of all 13 coding genes

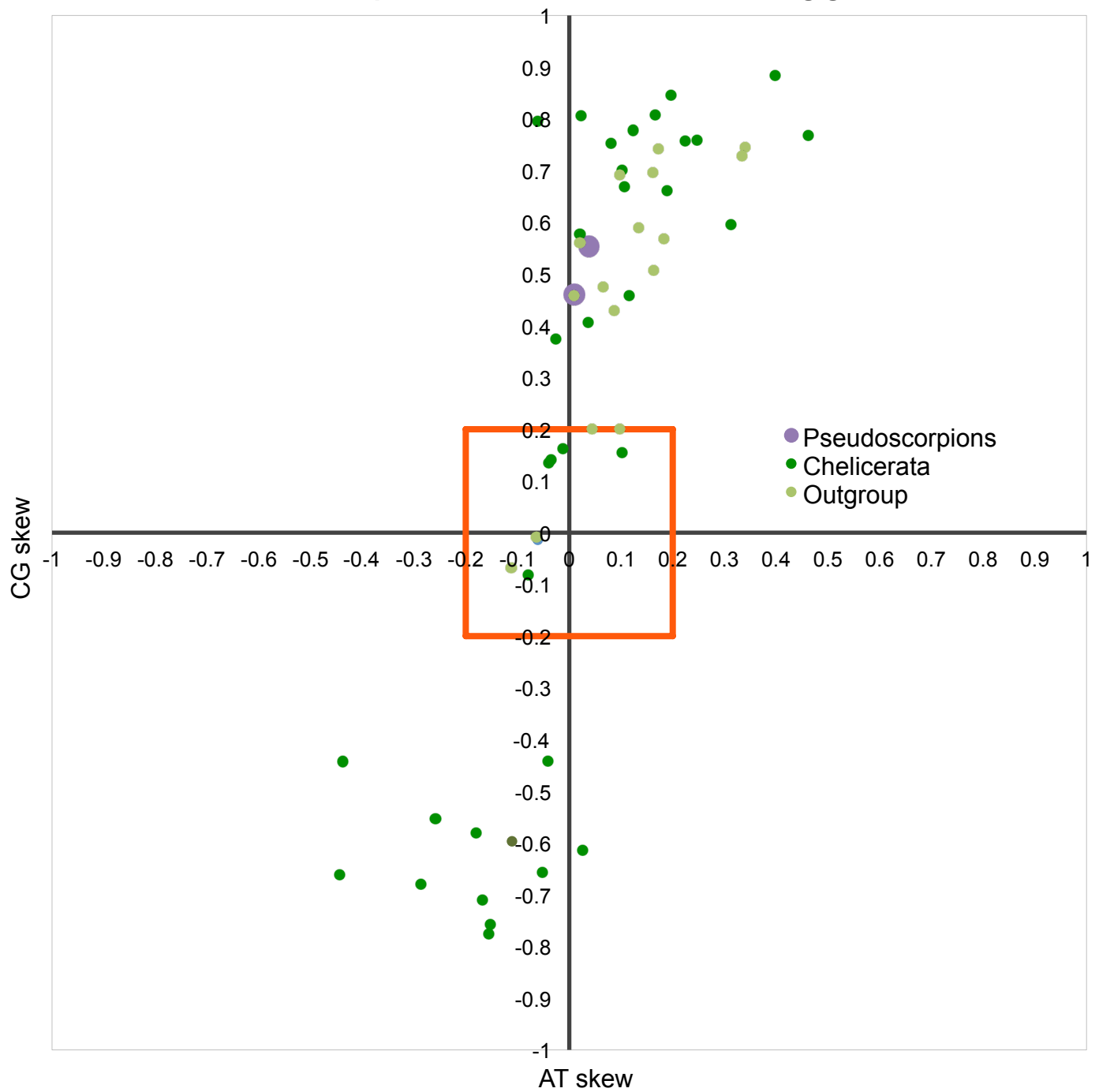

Supplement: Additional file 3 — Graph of nucleotide skew at 3rd codon positions of mitochondrial protein-coding genes of arthropods. CG skew is plotted along the Y-axis, and AT skew is plotted on the X-axis. Skew was calculated following Perna and Kocher [65], and described in the Methods. The red square bounds the taxa we considered to not have a pronounced CG or AT nucleotide skew, that is, within the range of -0.2 to +0.2. These taxa have purple branches leading to them in Figure 6. The two purple dots depict the pseudoscorpions, the dark green dots depict the other chelicerates, and the light green dots depict the arthropod outgroup taxa analyzed in this study. The taxa used to create this graph are given in Additional file 1. [file 1471-2148-12-31-S3.PDF]
