# Supplementary material for: Pseudoscorpion mitochondria show rearranged genes and genome-wide reductions of RNA gene sizes and inferred structures, yet typical nucleotide composition bias
Source: BMC Evol Biol. 2012 Mar 12;12:31. doi: 10.1186/1471-2148-12-31 (PMC3325882; doi:10.1186/1471-2148-12-31)
Supplement: Additional file 1 — List of taxa used in this study. List of taxa used in this study, arranged by their Linnaean taxonomy, with GenBank accession numbers for their mitochondrial genome sequence. [file 1471-2148-12-31-S1.PDF]

Additional file 1

Subphylum	Class	Subclass	Order	Suborder	Family	Subfamily	Species	GenBank No.
Chelicerata	Pycnogonida		Pantopoda		Tanystylidae		*Tanystylum orbiculare*	NC_014505
					Ammotheidae		*Ammothea hilgendorfi*	GU370075
							Achelia bituberculata	NC_009724
					Nymphonidae		*Nymphon unguiculatum-charcoti*	GU370076
							Nymphon gracile	NC_008572
	Merostomata		Xiphosura		Limulidae		*Limulus polyphemus*	NC_003057
							Tachypleus tridentatus	NC_012574
	Arachnida	Acari: Parasitiformes	Ixodida		Argasidae		*Ornithodoros moubata*	NC_004357
							Carios capensis	NC_005291
					Ixodidae	Haemaphysalinae	*Haemaphysalis flava*	NC_005292
						Ixodinae	*Ixodes hexagonus*	NC_002010
						Rhipicephalinae	*Rhipicephalus sanguineus*	NC_002074
			Mesostigmata	Dermanyssina	Ologamasidae		*Stylochyrus rarior*	NC_013474
					Varroidae		*Varroa destructor*	NC_004454
		Acari: Acariformes	Prostigmata		Trombiculoidea		*Ascoschoengastia* sp. TATW-1	NC_010596
							Walchia hayashii	NC_010595
							Leptotrombidium pallidum	NC_007177
					Unionicolidae	Unionicolinae	*Unionicola foili*	NC_011036
			Astigmata		Pyroglyphidae	Dermatophagoidinae	*Dermatophagoides farinae*	NC_013184
			Oribatida	Mixonomata	Steganacaridae		*Steganacarus magnus*	NC_011574
			Pseudoscorpiones		Feaelloidea		*Pseudogarypus banksi*	JQ040544
					Cheliferoidea		*Paratemnoides elongatus*	JQ040543
			Amblypygi		Phrynidae		*Phrynus* sp.	NC_010775
					Phrynichidae	Damoninae	*Damon diadema*	NC_011293
			Uropygi	Thelyphonida	Thelyphonidae		*Mastigoproctus giganteus*	NC_010430
			Araneae	Mygalomorphae	Nemesiidae		*Calisoga longitarsis*	NC_010780
					Theraphosidae		*Ornithoctonus huwena*	NC_005925
				Araneomorphae	Salticidae	Pelleninae	*Habronattus oregonensis*	NC_005942
					Nephilidae		*Nephila clavata*	NC_008063
					Hypochilidae		*Hypochilus thorelli*	NC_010777
				Mesothelae	Heptathelidae		*Heptathela hangzhouensis*	NC_005924
			Ricinulei				*Pseudocellus pearsii*	NC_009985
			Scorpiones		Buthidae		*Buthus occitanus*	NC_010765
							Centruroides limpidus	NC_006896
					Chactidae	Uroctoninae	*Uroctonus mordax*	NC_010782
			Solifugae		Eremobatidae		*Eremobates palpisetulosus*	NC_010779
					Ammotrechidae		*Nothopuga* sp.	NC_009984
			Opiliones	Palpatores	Phalangiidae	Phalangiinae	*Phalangium opilio*	NC_010766
Myriapoda	Chilopoda	Pleurostigmophora	Lithobiomorpha		Lithobiidae		*Lithobius forficatus*	NC_002629
					Ethopolyidae		*Bothropolys* sp. SP-2004	NC_009458
		Notostigmophora	Scutigeromorpha		Scutigeridae		*Scutigera coleoptrata*	NC_005870
	Diplopoda	Helminthomorpha	Spirobolida		Spirobolidae		*Narceus annularus*	NC_003343
			Spirostreptida		Harpagophoridae		*Thyropygus* sp. DVL-2001	NC_003344
			Julida		Nemasomatidae		*Antrokoreana gracilipes*	NC_010221
	Symphyla				Scolopendrellidae		*Symphylella* sp. YG-2006	NC_011572
					Scutigerellidae		*Scutigerella causeyae*	NC_008453
Hexapoda	Ellipura		Collembola	Poduromorpha	Poduridae		*Podura aquatica*	NC_006075
	Insecta	Neoptera	Diptera	Brachycera	Drosophilidae	Drosophilinae	*Drosophila yakuba*	NC_001322
				Nematocera	Culicidae	Anophelinae	*Anopheles gambiae*	NC_002084
			Hemiptera		Reduviidae	Triatominae	*Triatoma dimidiata*	NC_002609
Crustacea	Maxillopoda	Thecostraca	Pedunculata	Scalpellomorpha	Pollicipedidae		*Pollicipes polymerus*	NC_005936
	Malacostraca	Eumalacostraca	Decapoda	Pleocyemata	Paguridae		*Pagurus longicarpus*	NC_003058
	Branchiopoda	Sarsostraca	Anostraca		Artemiidae		*Artemia franciscana*	NC_001620
		Phyllopoda	Diplostraca	Cladocera	Daphniidae		*Daphnia pulex*	NC_000844
